# Brand loyalty in the face of stockouts

**DOI:** 10.1007/s11747-023-00924-8

**Published:** 2023-03-16

**Authors:** Uzma Khan, Alexander DePaoli

**Affiliations:** 1 Miami Herbert Business School, University of Miami, 5250 University Dr., Coral Gables, FL 33146 USA; 2 D’Amore-McKim School of Business, Northeastern University, 360 Huntington Ave, Boston, MA 02115 USA

**Keywords:** Brand loyalty, Stockouts, Shortage, Scarcity, Upgrades, Mood-Repair, Consumer-Expectations, Retailing, COVID-19, Substitution

## Abstract

An important managerial challenge is understanding consumers’ reactions to stockouts of a desired product―will they stay brand loyal or switch to competing brands? We posit that consumers are more likely to prefer substitutes from the same brand when a stockout is unexpected (vs. expected). This tendency arises as consumers feel greater negative affect upon encountering an unexpected stockout, which leads them to choose alternatives that provide greater affective value to ameliorate their negative feelings. Since the brand is a relatively affect-rich attribute compared to common non-brand attributes (e.g., price and quantity), consumers facing an unexpected stockout are more likely to choose a same-brand substitute. Five studies illustrate the effect and support the process by demonstrating that unexpected stockouts do not result in brand loyalty when non-brand attributes offer greater affective value than the brand. We further show that managers systematically mispredict how consumers’ expectations of stockouts relate to brand loyalty.

**Supplementary Information:**

The online version contains supplementary material available at 10.1007/s11747-023-00924-8.

Imagine that you are a marketing executive at a large company and are advised that the company is likely to have shortages of a specific product in the coming weeks, meaning that your target consumers might encounter stockouts of this product. However, your company offers several products in the category: for example, perhaps you sell paper products and only your regular 1-ply toilet paper is running low, or you sell bikes and only the base model is unavailable. Your goal is to maximize the likelihood that your customers, who may not be able to find their desired product, will choose to buy a substitute from among your other products rather than defect to a competitor’s product. In other words, your job is to ensure brand loyalty. In this regard, you now need to decide whether or not it is prudent to make public the information that product shortages are possible. What would you advise in order to maximize the likelihood of retaining customers? Would you inform them of potential shortages or let them be surprised if they encounter a stockout?

Product shortages can commonly arise due to demand shocks, production hiccups, supply-chain disruptions, and capacity constraints. How consumers respond to these shortages is an issue of longstanding importance in business research and practice. A critical question for managers interested in adapting their commercial strategies or forecasting demand in the face of such shortages is how to best ensure that consumers will be more likely to stay brand loyal when faced with product stockouts, particularly if they have the opportunity to switch to other brands in pursuit of a better deal. The importance of addressing this question was highly salient when the COVID-19 pandemic slammed the brakes on much of the global supply chain beginning in early 2020, resulting in unprecedented stockouts (Cavallo & Kryvtsov, 2021; Tariton, 2020) in health-related products (e.g., hand sanitizer, soap, and masks) as well as in everyday consumer products (e.g., toilet paper, canned goods, and flour) and in discretionary spending (e.g., dumbbells, hair clippers, and video game consoles). As consumers faced stockouts, firms faced a pressing challenge to rapidly forecast consumers’ reactions to such stockouts. The reports from consumer experts were mixed: some indicated that consumers were staying loyal to their favorite brands and were willing to pay more for them, while others reported rising instances of brand switching, suggesting shifts to lower-priced brands (Becdach et al., 2020; Ju & Jang, 2023; Klein, 2020).

In this article, we provide a framework to predict when product shortages and stockouts may engender brand loyalty and when they may lead to brand switching. We posit that one factor that determines if consumers stay brand loyal following a stockout is whether or not they expected that the product could be out-of-stock. We reason that consumers feel greater negative affect when encountering an unexpected (vs. expected) stockout, which in turn makes them more likely to choose an alternative option that provides greater affective value in order to ameliorate or otherwise repair their negative feelings. We argue that the brand is a relatively more affect-rich product attribute compared to common non-brand attributes such as price and quantity (Grisaffe & Nguyen, 2011; Keller, 1993; Kim & Sullivan, 2019; Thomson et al., 2005), and therefore consumers faced with an unexpected (vs. expected) stockout of a desired product should be more likely to stay brand loyal even when such a decision comes at a cost of a higher price or smaller quantity. We show support for this proposition in five studies, and further explore when such brand loyalty may or may not arise as a function of our novel affect-driven mechanism.

To illustrate the managerial necessity of our findings, we posed the opening question to 735 executives with an average of 25 years’ experience, and invited them to advise this hypothetical large company striving to ensure that they did not lose customers to rivals as a result of upcoming stockouts. They were asked whether this goal of brand loyalty was best served by making the knowledge of potential shortages public (thus giving customers an expectation of stockouts) or by withholding this information (thus ensuring no expectation of stockouts). Overwhelmingly, respondents recommended informing customers of potential stockouts (80.30%), showing that their intuition is opposite to how we observe that expectations of stockouts actually influence brand loyalty. Furthermore, their advice did not improve with experience, but instead got worse. We also presented the same survey to a novice population (212 workers on Amazon Mechanical Turk) and found that their intuition was similar to that of the executives (89.15% recommended informing customers of the potential stockouts). Thus, both expert managers and novices alike incorrectly predict that informing customers about potential stockouts is the best way to keep them brand loyal (details of these studies are provided in Appendix A). The fact that the experts, who have training in business and have significant professional experience, are as poor as novices at anticipating the effect of expectations on brand loyalty underscores the managerial importance and relevance of the current finding. Given our claims, we would generally advise a manager facing the opening scenario to avoid informing customers of potential stockouts in advance. This way, consumers will not be expecting the stockouts and will be more likely to choose a same-brand substitute for the out-of-stock item. We further discuss the novel managerial insights arising from our findings in the General Discussion.

Our work contributes to the literatures on scarcity, substitution, and branding decisions. Past research on scarcity has shown that consumers tend to pick the most similar substitute available to them when their desired product is out-of-stock (e.g., Arens & Hamilton, 2016). However, this extant literature is silent about the attribute(s) that consumers prefer to be similar between the stocked-out option and the substitute, and by extension, does not generate any insight into the managerially critical question of whether, and by what mechanism, a general desire for similarity can be leveraged into a preference for the same brand, i.e., brand loyalty. Our research provides a novel synthesis of the literatures on expectations and affect-driven consumption to fill this void and to provide a mechanism not only for when stockouts lead to brand loyalty, but for suggesting a new perspective on when consumers may or may not show the aforementioned preference for similarity. In doing so, our work further contributes to prior research on branding by assessing brand loyalty behaviors and positing both a novel antecedent–i.e., expectations of stockouts–and a novel mechanism–i.e., affect–for brand loyalty. Our findings also have implications for substitution decisions more broadly than our substantive topic of brand loyalty, which we discuss in the General Discussion. Finally, we also contribute to the rapidly developing research on how global trade disruptions, such as those caused by pandemics, impact corporate practices (Das et al., 2021) and consumer behavior (e.g., Friedman & Toubia, 2022; Kwon et al., 2022).

## Theoretical background

An important managerial challenge is understanding how consumers respond to product stockouts: are they motivated to stay brand loyal or are they likely to switch to competing brands when their desired product is unavailable? Understanding the factors that drive brand loyalty is critical for businesses given that a firm’s brand equity is inextricably linked to the loyalty of its customers (Aaker, 1991; Borkovsky et al., 2017; Hemsley-Brown, 2022; Khamitov et al., 2019). Consumers are considered brand loyal when they make consistent repurchases of a brand’s products owing to a positive affinity for the brand (Day, 1969). Thus, loyalty is not only the behavioral response of repeat purchase, but also an attitudinal response (Jacoby & Chestnut, 1978; Mellens et al., 1996). If consumers make their purchase decisions with little concern for the brand name and instead prioritize non-brand features, such as price or quantity, there is likely little to no brand loyalty. Thus, a useful approach to capturing brand loyalty is to consider consumers’ likelihood to switch brands when the focal brand makes a change to its price or features (Aaker, 1991; Liu-Thompkins et al., 2022; Swaminathan et al., 2020). Product stockouts provide a particularly important context in which to study consumer loyalty as stockouts require consumers to decide what to buy when a preferred product from the brand is rendered unavailable, thus leaving them potentially choosing between a different product from the same brand and alternative products from competing brands. Note that the context we are examining is not one in which consumers are confronted with a singular option therefore must accept whatever is available. Instead, our focus is on how consumers choose among multiple available alternatives, including options from the same brand, when the product they wanted is stocked-out or otherwise unavailable. We believe that these contexts are more common and important in a modern consumer economy, and more relevant to the consumer experience as many do seek out or otherwise judge products on the basis of brand. In such scenarios, companies would naturally hope that their customers’ brand loyalty is strong enough to keep them from switching to a competitor even if such loyalty incurs additional costs in terms of non-brand features (e.g., higher price, lower quantity, or greater inconvenience relative to an alternative).

While no prior research has directly examined the impact of stockouts on brand loyalty, research on product scarcity provides some guidance (for review, see Hamilton et al., 2014, 2019). A relevant finding in this extant research is that product scarcity magnifies desirability and can increase both the perceived value of, and the demand for, a scarce product (Gierl & Huettl, 2010; Inman et al., 1997; Kristofferson et al., 2017; Lynn, 1991; Parker & Lehmann, 2011; van Herpen et al., 2009). For example, Worchel et al. (1975) showed that cookies in short supply were desired and valued more than cookies in abundant supply. In another study, books with limited availability due to market circumstances were deemed more unique than abundantly available books (Verhallen & Robben, 1994). Moreover, if consumers feared that a grocery store might start running low on their favorite yogurt, they were more likely to buy the flavor(s) that they liked the most (Zhu & Ratner, 2015). In comparing message effectiveness, Aggarwal et al. (2011) found that messages stressing limited quantities (i.e., the potential of a shortage) were more persuasive than those stressing limited time. One reason that product scarcity and stockouts increase the desirability of a scarce product is that they engender negative feelings such as reactance (Clee & Wicklund, 1980; Fitzsimons, 2000; Worchel et al., 1975) and jilting (Litt et al., 2010), which in turn motivate greater desire for the stocked-out product as a means to counteract these negative feelings. Increased desirability of the stocked-out product is also shown to influence consumers’ choice of substitutes such that consumers are drawn to pick substitutes that most closely resemble an out-of-stock option to fill the void left by the denied product with something similar to it (Arens & Hamilton, 2016; Litt et al., 2010). This occurs even when more dissimilar substitutes would better fulfill consumers’ consumption goals and result in greater satisfaction (e.g., Arens & Hamilton, 2016; Huh et al., 2016).

The scarcity literature, in sum, predicts that consumers prefer alternatives that are similar to stocked-out products. This prediction, however, is limited in its theoretical and applied scope because products can be similar or dissimilar on multiple dimensions, and it does not shed light on the dimensions on which consumers will seek similarity. For example, would consumers prefer a similar brand or a similar price? And, more critically, when faced with tradeoffs between similar and dissimilar attributes, would they pay higher price for a similar brand or would they prioritize a similar price and go for a different brand? This gap begs a critical question: when and why are consumers more likely to choose an alternative from the same brand as the originally desired product versus a product that is instead similar on important non-brand features, such as price or quantity? We provide a framework to determine when stockouts may or may not lead to brand loyalty and posit that this hinges on two key factors discussed next: whether the stockout is expected or unexpected, and the relative affective value of the brand as compared to other key attributes.

### Expectations of stockouts and affective value of a brand

Expectations play a key role in the formation of affective reactions. Research has shown that individuals’ affective reactions are formed once an experience is deemed to be consistent or inconsistent with their expectations, and the direction and salience of the discrepancy between expectations and the actual outcome can determine the valence and intensity of such affective reactions (e.g Geers & Lassiter, 1999; Wilson et al., 1989). For example, if a consumer sees a film that they expect to be good, but which is instead inarguably bad, they will tend to have a stronger negative reaction than if they had no initial expectation of the film’s quality (Geers & Lassiter, 1999). Likewise, when consumers are denied an outcome that they believe was owed to them or which was otherwise expected, they tend to experience more intense and more maladaptive frustration (e.g., van Steenburg et al., 2013). In light of this, we claim that negative affective responses to stockouts should be viewed as a consequence of expectation disconfirmation: if consumers expect to be able to purchase a product but instead discover that they cannot because it is out-of-stock, that violation of their expectation should lead to greater negative affect than if they had expected the possibility of a stockout. While the extant scarcity literature has observed that negative affect can drive a preference for similarity via the specific experiences of reactance or jilting (i.e., Clee & Wicklund, 1980; Fitzsimons, 2000; Litt et al., 2010), we argue that such research has overlooked a more fundamental influence of negative affect on consumers’ decision-making, which leads to our second claim: consumers experiencing greater negative affect due to an unexpected stockout should be more likely to seek products or attributes that are intended to ameliorate or otherwise repair those negative feelings. This second notion is consistent with research showing that shifts in consumers’ affective states can trigger a regulatory system intended to manage their mood, which influences subsequent decision-making such that greater deviation from an ideal affective state leads to stronger countervailing influences on behavior (Andrade, 2005). For example, consumers in a negative (vs. positive) mood are more likely to help others in order to counteract their own negative moods (Cialdini et al., 1973), are more likely to indulge in high-calorie snacks in hopes of increasing their happiness (Labroo & Mukhopadhyay, 2009; Tice et al., 2001), and are more likely to buy products that provide an opportunity to improve their circumstances (Lerner et al., 2004). To summarize, our two claims suggest that consumers who experience more severely violated expectations in the form of an unexpected stockout will display stronger preferences for substitutes with affect-rich attributes that can better counterbalance their more acute negative affect. We next explain how this response to an unexpected stockout would lead to brand loyalty.

We propose that the brand is frequently not only a salient point of comparison between two products for the purpose of assessing similarity, but it is also a relatively more affect-rich attribute than many other salient non-brand attributes. It is well established that product attributes vary in terms of their affective value to the consumer, most notably those relating to hedonic enjoyment or symbolic meaning (Alba & Williams, 2013; Batra & Ahtola, 1991; Khan et al., 2005). Brands have been shown to deliver affective value to consumers as a function of being the focal point both for consumers’ beliefs about products or companies and for their emotional attachments to them (Grisaffe & Nguyen, 2011; Keller, 1993; Kim & Sullivan, 2019). For this reason, consumers are apt to form affect-laden relationships with brands (Aggarwal, 2004; Fournier, 1998; Kleine et al., 1993; Loureiro et al., 2012) and to project onto them their own identities (Berger & Heath, 2007; Chernev et al., 2011) and status (Braun & Wicklund, 1989; Thomson et al., 2005). In contrast, many common non-brand attributes like price and quantity tend to carry relatively little affective value in normal market contexts. Therefore, we posit that if a consumer experiences an unexpected stockout (and hence has a more intensely negative experience), then substitute products sharing the brand of the out-of-stock product should become more attractive as they will provide greater affective value and thus will be more appropriate for counterbalancing the consumer’s negative affective experience.

Our proposed affect-based process naturally suggests a relevant boundary condition, namely that if the brand is not the most affect-rich attribute in the choice of substitutes, then the consumer will be less inclined to choose on the basis of the brand. This leads us to predict that if the underlying need for affective value can be better satisfied with an affect-rich, non-brand attribute (e.g., that provides aesthetic or hedonic pleasure), then we would not expect to observe brand loyalty following an unexpected stockout. Thus, we provide a mechanism that not only predicts when and why consumers will stay brand loyal in the face of a stockout, but that also makes novel predictions about when and why consumers may or may not display the preference for similar substitutes observed in prior scarcity research. Our claims are distinct from scarcity research not only because that literature does not make predictions about which similar attributes a consumer will prefer, but also because we suggest that a preference for the same brand arises not simply out of a desire for similarity but from brands’ ability to deliver affective value to counter the negative affect generated by unexpected stockouts.

### Current research

We formally hypothesize that:

#### H1

Consumers who are less likely to expect a product stockout (vs. those who are more likely) will be more likely to demonstrate brand loyalty in their choice of substitutes following the stockout.

#### H2

Consumers who do not expect product stockouts (vs. those who do) will experience greater negative affect in the face of a stockout, which will in turn lead to greater brand loyalty.

#### H3

Unexpected stockouts should not result in greater brand loyalty when non-brand attributes offer greater affective value than the brand.

To clarify these hypotheses, it is important to elaborate on the conceptualizations and operationalizations of negative affect, expectations, and brand loyalty that we use in our empirical tests.

#### Negative affect

To operationalize the negative affective experience associated with stockouts, we focus on the emotion of frustration. We take this approach because frustration is a negative emotion that is specifically associated with the failure to attain a desired outcome (Gelbrich, 2010; Smith & Ellsworth, 1985). Frustration arises when an anticipated reward or experience is blocked due to internal (e.g., person’s lack of skills or knowledge) or external (e.g., environmental or situational) factors (Shorkey & Crocker, 1981; van Steenburg et al., 2013). A stockout is an example of such a block in the consumer context, making frustration a highly relevant negative emotion for our research. Furthermore, frustration is well-suited to cleanly capture negative affect in response to stockouts for two important reasons. First, it tends to be highly correlated with other negative emotions, such as anger or irritation, that make up consumer reactions to undesirable shopping experiences, and has thus been shown to be sufficiently universal and relatable that it can reliably capture our negative affective reactions of interest (Wetzer et al., 2007). Second, feelings of frustration in consumer contexts are associated not only with negativity but with a motivation to resolve that negativity (Stauss et al., 2005; van Steenburg et al., 2013), making the construct well-suited to capture the general negative affect that drives consumers to seek similarity following a stockout.

#### Expectations

Consumers’ expectations of future events are based on the ease with which they can recall or conceptualize examples of such an event such that greater ease leads to greater beliefs that the event will occur or reoccur (Miller, 1998; Oskarsson et al., 2009; Tversky & Kahneman, 1974). For example, investors tend to judge the potential of an investment based on information that was recently in the media rather than based on all relevant facts (Tversky & Kahneman, 1974) and consumers base predictions of the likelihood of product failure on how easily they can recall incidences of such failures (Folkes, 1988). Thus, the more cognitively available an event is to a consumer, the more they are likely to expect it. Therefore, we conceptualize higher expectations of a stockout in terms of how available that stockout is to a given participant, and we capture such expectations in several ways. In Study 1, consumers’ personal prior experiences with stockouts serve as the measure of their expectations of stockouts. In Studies 2 and 3, we leverage lay intuitions about how stockouts may be driven by increased demand to capture consumers’ expectations of stockouts. In Study 4, we manipulate the availability of a possible stockout by whether we explicitly warn participants about product shortages. In Study 5, we directly measure consumers’ expectations prior to them learning about a stockout. These different approaches help us to develop a theoretically and practically relevant triangulation of the expectations construct.

#### Brand loyalty

Brand loyalty can manifest in several ways. As a conservative test, we only examine decisions in which participants must incur a cost in order to demonstrate brand loyalty, generally by making a tradeoff between brand and price or a tradeoff between brand and quantity. We mainly focus on price and quantity tradeoffs as these are important non-brand attributes in most choice contexts, so it is relevant to understand how consumers tradeoff these attributes versus brand. Hence, we test our predictions in decision contexts in which the brand loyal option is either an upgrade (entailing greater price) or a downgrade (entailing smaller quantity). In the upgrade decisions, consumers demonstrate brand loyalty by choosing a more expensive substitute from the same brand as the stocked-out product rather than switching to a substitute which is similarly priced to the stocked-out product but from a different brand. Such upgrade decisions allow us to investigate when brands may be able to take advantage of consumers’ preference for similarity not only to retain customers during stockouts, but to upsell to them. In the downgrade decisions, consumers demonstrate brand loyalty by choosing a substitute that offers a smaller quantity from the same brand as the stocked-out product rather than switching to a substitute that offers the same quantity as the stocked-out product but from a different brand.

#### Overview of the studies

We next report five studies in support of our hypotheses (see Appendix B for a summary of these studies). The first two studies were run during May of 2020 when the COVID-19 pandemic had caused widespread shortages, thus allowing us to explore brand loyalty in the face of stockouts by using consumers’ own experiences of pandemic-related stockouts (Study 1) as well as their beliefs about how COVID-19 had impacted demand for various categories (Study 2). Next, we broaden the discussion to commonplace market contexts in which expectations of product shortages are measured (Studies 3 and 5) and manipulated (Studies 3 and 4). We also examine real choices (Study 4) and explicitly test our proposed affect-based mechanism against alternative accounts (Study 5).

## Study 1: Experience of shortage during COVID-19

Study 1 tested the prediction that brand loyalty is more likely to arise when consumers do not anticipate their desired product to be out of stock (H1), and that this effect is driven by greater negative affect at the stockout due to the event not being anticipated (H2).

To examine consumers’ expectations of product stockouts, we looked at participants’ own real experiences of encountering stockouts during the COVID-19 pandemic. In this context, we examined their willingness to trade off price for brand loyalty when faced with a stockout of a desired product by having them choose a substitute from between a similarly priced product from a different brand and an expensive upgrade product from the same brand. We posit that those who had not experienced a stockout (vs. those who had) would be more likely to upgrade and stay brand loyal even if that meant paying more to do so (H1), and that this brand loyalty would be driven by greater negative affect experienced upon encountering the unexpected (vs. expected) stockout (H2).

### Method

U.S.-based Amazon Mechanical Turk (MTurk) workers (N = 303; *M*_age_ = 37.2, 39.9% female) completed the study in May 2020 during the product shortages of the early COVID-19 pandemic. Stimuli were a list of nine product categories that had appeared in media stories about COVID-19 stockouts (e.g., Tariton, 2020). The study used a single factor between-subjects design with repeated measures such that each participant completed a stockout task (within-subject) for three categories randomly selected from the list, giving us a total of 909 observations. Product categories included: toilet paper, hand sanitizer, hand soap, rice, chocolate, running shoes, laundry detergent, televisions, and headphones.

For each category to which participants were assigned, they first indicated whether or not they had a preferred brand in that category. This allowed us to control for strong idiosyncratic preferences, which could result in greater frustration upon encountering a stockout independent of the effect of expectations. Next, participants indicated if they had experienced a stockout in that category in the preceding two months. They were then asked to imagine their next shopping trip for a product from the given category. Participants who indicated a preferred brand were told that the specific product from this brand which they had intended to buy was out-of-stock. Participants who did not have a preferred brand were told that an unspecified brand and product which they had intended to buy was out of stock. As a measure of negative affect, all participants then rated how frustrated they would be to encounter such a stockout (1 = Not at all to 7 = Extremely). Next, all participants chose a substitute from among three options: a similarly priced product from a different brand (*D*), an upgraded higher priced product from the same brand (*S* +), or an upgraded higher priced product from a different brand (*D* +). This final option was included to rule out the possibility that a generalized preference for upgraded options could be driving the effect.

Participants then completed a battery of 12 questions about the emotions they felt toward their lives during the pandemic. Responses did not interact with our primary analyses, and so are not discussed further. Lastly, participants completed a demographic questionnaire. We controlled for these factors in all analyses (see materials in Appendix C).

### Results and discussion

Participants had a preferred brand in 42.57% of product categories and reported having experienced a stockout in 33.44% of the categories. As predicted, a multinomial logistic regression found that prior experience with a stockout led to a significantly lower likelihood of choosing *S* + over both *D* (*b* = -0.80, *p* < 0.0001) and *D* + (*b* = -1.01, *p* < 0.002; choice shares in Table 1). However, while there was no difference in the choice of *D* + over *D* (*b* = 0.21, *p* = 0.457; see Model 1-Table 2), thus ruling out the possibility of a generalized shift toward upgrade options driving the effect. Given our focus on brand loyalty, in the remaining analyses we collapsed the results for *D* and *D* + in order to focus on choice of *S* + as the dependent variable (e.g., Neumann et al., 2016).

**Table 1:** Study 1 results by product category

	Had a preferred brand (%)	Experienced a stockout (%)	Frustration	Choice share (%)		
				*D*	*D* +	*S* +
Toilet paper	50.96	75.00	4.68 (1.80)	73.08	12.50	14.42
Hand sanitizer	21.88	66.67	4.04 (2.11)	78.13	7.29	14.58
Hand soap	41.18	43.14	3.95 (1.96)	82.35	6.86	10.78
Rice	20.59	38.24	3.92 (1.98)	80.39	9.80	9.80
Chocolate	48.99	27.55	3.87 (1.87)	76.53	4.08	19.39
Running shoes	58.09	10.48	4.32 (1.88)	57.14	3.81	39.05
Laundry detergent	52.63	21.05	4.04 (1.86)	70.53	9.47	20.00
Televisions	45.19	6.73	4.14 (1.80)	62.50	4.81	32.69
Headphone	43.00	13.00	3.97 (1.75)	60.00	6.00	34.00

Participants indicated if they had a preferred brand, had experienced a stockout in the product category during COVID-19, and their level of frustration at the stockout. Choice shares reflect choice of a same priced substitute from a different brand (*D*), a more expensive substitute from a different brand (*D* +), or a more expensive substitute from the stocked-out brand (*S* +)

**Table 2:** Study 1 regressions model results

Model Number:		1			2	3	4	5
Model Type:		Multinomial logistic			Binomial logistic	Linear	Binomial logistic	Binomial logistic
DV:		Choice of *S* + (reference *D*)	Choice of *D* + (reference *D*)	Choice of *S* + (reference *D* +)	Choice of *S* +	Frustration	Choice of *S* +	Choice of *S* +
Predictors								
Experienced stockout	-0.80*** (0.20)		0.21 (0.29)	-1.01** (0.32)	-0.70*** (0.21)	0.98*** (0.12)		-0.93*** (0.22)
Brand preference	1.23*** (0.18)		0.31 (0.28)	0.92** (0.00)	1.42*** (0.19)	0.78*** (0.11)	1.18*** (0.19)	1.27*** (0.20)
Frustration	0.23*** (0.05)		0.12 (0.08)	0.11 (0.19)			0.17** (0.05)	0.23*** (0.06)
Age	-0.01 (0.01)		0.01 (0.01)	-0.02 (0.01)	-0.01 (0.01)	0.00 (0.01)	-0.01 (0.01)	-0.01 (0.01)
Gender (female)	-0.57** (0.19)		-0.14 (0.28)	-0.43 (0.17)	-0.51* (0.21)	0.36* (0.16)	-0.57** (0.21)	-0.61** (0.21)
Employed	-0.10 (0.09)		-0.04 (0.13)	-0.06 (0.67)	-0.13 (0.10)	-0.12 (0.08)	-0.08 (0.10)	-0.10 (0.10)
English	-0.70 (0.51)		-0.39 (0.80)	-0.31 (0.71)	-0.87 (0.58)	-0.89^•^ (0.51)	-0.77 (0.56)	-0.71 (0.58)
Income	-0.00 (0.00)		-0.04* (0.00)	0.01 (0.12)	-0.00 (0.00)	0.00 (0.00)	-0.00 (0.00)	-0.00 (0.00)
Urban	-0.05 (0.13)		0.67** (0.00)	-0.71** (0.00)	-0.12 (0.15)	-0.02 (0.12)	-0.09 (0.15)	-0.12 (0.15)
Intercept	-2.42** (0.81)		-0.58 (1.21)	-1.84 (1.31)	-1.50^•^ (0.83)	6.29*** (0.65)	-1.08 (0.76)	-2.99** (0.91)
AIC:			1294.09		878.87	3552.22	879.72	861.98

*N* = 909 observations. Standard errors in parentheses. Degrees of freedom for mixed-models calculated using the Satterthwaite Approximation. Significance levels: *p* < .001***, *p* < .010**, *p* < .050*, *p* < .100^•^

We conducted a series of mixed-model regressions to test our predictions, controlling for: within-participant variance, whether or not participants indicated a preferred brand in the category, and demographic variables (degrees of freedom were calculated using the Satterthwaite approximation; see Table 2 for regression models). In support of H1, a binomial logistic regression found that lower expectation of stockouts indeed predicted the choice of substitute such that participants who had not experienced shortages tended to prefer the *S* + option 23.71% of the time versus 17.82% of the time when they had experienced shortages (*b* = -0.70, *z*(889) = -3.35, *p* < 0.001, odds ratio = 2.01; Model 2-Table 2).

Using the same controls as noted above, linear regressions found that prior experience with stockouts predicted frustration such that participants reported greater frustration when they had not experienced a stockout (*M* = 4.88, SE = 0.10) relative to when they had (*M* = 3.72, SE = 0.08; *b* = 0.98,* t*(806) = 8.51, *p* < 0.001, *d* = 0.65; Model 3-Table 2). This provides initial evidence showing that consumers experience less (more) negative affect with a stockout when they expect (do not expect) the possibility of such a stockout. Furthermore, in support of H2, we found that greater frustration predicted greater preference for *S* + (*b* = 0.17, *z*(798) = 3.26, *p* < 0.002, odds ratio = 1.19; Model 4-Table 2). The H2 pathway, whereby prior experience with stockout leads to lower likelihood of upgrade behavior through frustration, was tested using a bootstrapped mediation analysis with 5000 iterations (coefficients standardized per MacKinnon & Dwyer, 1993), found evidence for a significant indirect effect (bias corrected 95% CI [0.47, 1.56]; see Model 5-Table 2).

The results support the proposition that consumers are more likely to show brand loyalty when a stockout is unexpected (vs. expected) due to prior experience with stockouts. Note that our results cannot be explained by possible budget or resource constraints arising during the pandemic (e.g., lower income due to job losses), which would predict that consumers should not prefer to upgrade regardless of their prior experience of shortage. The effect also cannot be explained simply by increased attraction of the products due to scarcity, which would predict either that consumers should be equally likely to upgrade when faced with a stockout regardless of their prior experience of shortage or that they should be more (rather than less) likely to upgrade when they had experienced the shortage in the past.

## Study 2: Expectation of increased demand during COVID-19

Whereas Study 1 made use of participants’ own experience of product shortages to capture their expectations of future stockouts, Study 2 used product categories in which, owing to the specific impacts of COVID-19, participants would expect increased demand and corresponding shortages. This approach meets two objectives. First, using pre-identified products that vary on expectations of increased demand addresses a concern that not all participants have personal experience of shortage in all categories. Second, consumers may form expectations of shortages due to factors other than their own experiences, such as media coverage or the experiences of others. This approach provides a way to capture such second-hand expectations.

### Method

U.S.-based MTurk workers (N = 674; *M*_age_ = 36.0, 49.7% female) completed the study during the COVID-19 pandemic. The study used a single factor between-subjects design with repeated measures as in Study 1. Each participant was presented with three product categories randomly pulled from a list of 13 products (toilet paper, hand sanitizer, hand soap, laundry detergent, running shoes, bicycles, chocolate, televisions, ice cream, water filters, water pitchers, coffee makers, and headphones), giving us a total of 2202 observations.

As in Study 1, participants indicated for each category whether they had a preferred brand, and then imagined a shopping trip in which they encountered a stockout, rated their frustration, made a substitution choice (between *D*, *D* + , and *S* +), and completed questions about demographics and general COVID-related emotions (see materials in Appendix C).

#### Demand and shortage perceptions across different product categories

This study used products for which participants had varying expectations of demand increase and shortage likelihood due to COIVD-19. A separate set of U.S.-based MTurk workers (N = 102; *M*_age_ = 39.3, 46.8% female) were shown 30 products (listed in Table 3) in a randomized order and asked to indicate whether they agreed or disagreed that the COVID-19 pandemic had increased demand for each. The categories were then coded based on the proportion of respondents agreeing that demand had increased during COVID-19: if the proportion was significantly greater than 50%, it was coded as “high expectations of demand increase”; significantly less than 50% was coded as “low expectations of demand increase”; and if the proportion was no different from 50%, it was coded as “unaffected expectations.” The results confirmed that toilet paper, hand sanitizer, hand soap, and laundry detergent were categories for which our population agreed that COVID-19 increased demand; running shoes and bicycles were categories for which our population disagreed that COVID-19 increased demand; and chocolate, televisions, ice cream, filter water pitchers, coffee makers, and headphones were categories for which there was no consensus that COVID-19 impacted demand (see Table 3). To confirm that expectations of demand increase related to COVID-19 reflect higher expectations of a shortage, we conducted another survey with members of the same population (N = 104, *M*_*age*_ = 39.1, 40.4% female). Participants indicated whether they agreed with the proposition that many products had been stocked-out during the initial months of COVID-19 due to consumers buying more of those products (1 = Strongly disagree to 5 = Strongly agree). As expected, participants indeed agreed with this statement as evidenced by the response mean being significantly greater than the scale midpoint (*M* = 4.18, SE = 0.06; *t*(103) = 18.55, *p* < 0.001).

**Table 3:** Study 2 product categories and expectations of demand increase

Product category	Agree that demand has increased due to COVID-19 (%)
Chocolate	57
Ice-Cream	52
Laundry Detergent	71
Sanitizer	94*
Toilet Paper	92*
Soap	92*
Bug Spray	34*
Cars	11*
Bikes	38*
Make-up	8*
Printer Paper	35*
Soda	55
Gasoline	14*
Bread	74.5*
Cough Medicine	68*
Bug Spray	34
Dog Food	50.5
Moisturizer	43
Running Shoes	32*
Bike	38*
Headphones	48
Coffee Makers	54
Televisions	51
Water Pitchers	46
Gym membership	8*
Cars	11*
Umbrellas	10*
Picture Frames	21*
Cloth Iron	14*
Dress Shoes	9*
Thermometers	75.5*
Bread Makers	57
Pianos	25*

^*^Proportions differing from 50% at *p* < .05

### Results and discussion

Each of the 13 product categories were coded according to whether their demand was seen to have increased, remain unaffected, or decreased due to COVID-19 as per the results in Table 3. In categories with higher expectation of demand increase due to COVID-19, *S* + was preferred 16.32% of the time, versus 27.11% of the time in categories with no change in expected demand, and 32.84% of the time in categories with low expectation of demand increase due to COVID-19 (full choice shares in Table 4).

**Table 4:** Study 2 results by product category

	Had a preferred brand (%)	Frustration	Choice share (%)		
			*D*	*D* +	*S* +
Toilet paper	50.58	3.51 (1.94)	79.41	10.00	10.59
Hand sanitizer	27.81	3.26 (1.98)	83.43	7.10	9.47
Hand soap	55.42	3.63 (1.96)	75.90	3.01	21.08
Laundry detergent	64.50	4.14 (1.87)	72.78	2.96	24.26
Running shoes	68.02	4.57 (1.90)	51.74	6.98	41.28
Bicycles	26.63	4.01 (1.86)	71.01	4.73	24.26
Chocolate	59.17	3.63 (1.79)	71.01	4.14	24.85
Televisions	50.30	4.31 (1.75)	61.08	8.38	30.54
Ice cream	61.08	3.62 (1.93)	70.06	8.98	20.96
Water filter pitchers	37.35	3.77 (1.94)	71.08	6.02	22.89
Coffee makers	39.64	4.07 (1.72)	66.27	4.73	28.99
Headphones	41.42	4.20 (1.86)	62.13	2.96	34.91

Participants indicated whether they had a preferred brand, had experienced a stockout in the product category during COVID-19, and their level of frustration at the stockout. Choice shares reflect choice of a same priced substitute from a different brand (*D*), a more expensive substitute from a different brand (*D* +), or a more expensive substitute from the stocked-out brand (*S* +)

Replicating Study 1, a multinomial logistic regression found that perceptions of increased demand led to a significantly lower likelihood of choosing *S* + over both *D* (*b* = -0.48, *p* < 0.0001) and *D* + (*b* = -0.44, *p* < 0.006) while there was no difference in the choice of *D* + over *D* (*b* = -0.05, *p* = 0.744; see Model 1-Table 5).

**Table 5:** Study 2 regressions model results

Model number:	1				2	3	4		5	
Model type:	Multinomial logistic				Binomial logistic	Linear	Binomial logistic		Binomial logistic	
DV:	Choice of *S* + (reference *D*)	Choice of *D* + (reference *D*)	Choice of *S* + (reference *D* +)		Choice of *S* +	Frustration		Choice of *S* +		Choice of *S* +
Predictors										
COVID-relevant	–0.48*** (0.09)	–0.05 (0.15)	–0.44** (0.16)		–0.65*** (0.09)	–0.36*** (0.05)				–0.54*** (0.10)
Brand preference	1.27*** (0.14)	0.50* (0.22)	0.77** (0.24)		1.92*** (0.14)	1.49*** (0.07)		1.20*** (0.15)		1.30*** (0.15)
Frustration	0.53*** (0.04)	0.43*** (0.06)	0.10^•^ (0.07)					0.55*** (0.05)		0.53*** (0.05)
Age	0.00 (0.00)	–0.02* (0.01)	0.02* (0.01)		0.00 (0.01)	0.00 (0.00)		0.00 (0.01)		0.00 (0.01)
Gender (female)	0.06*** (0.12)	–0.49* (0.21)	0.54* (0.22)		0.15 (0.14)	0.22* (0.11)		0.11 (0.14)		0.08 (0.14)
Employed	–0.13 (0.07)	–0.06 (0.12)	–0.08 (0.12)		–0.10 (0.08)	0.01 (0.06)		–0.15^•^ (0.08)		–0.14^•^ (0.08)
English	0.28 (0.36)	-0.73^•^ (0.44)	1.01* (0.51)		0.40 (0.39)	0.12 (0.28)		0.46 (0.41)		0.43 (0.41)
Income	0.00 (0.00)	-0.01^•^ (0.00)	0.01** (0.00)		0.00 (0.00)	–0.00 (0.00)		0.00^•^ (0.00)		0.00^•^ (0.00)
Urban	–0.10 (0.09)	0.42** (0.16)	–0.51** (0.16)		–0.19^•^ (0.10)	–0.06 (0.08)		0.16 (0.10)		–0.14 (0.11)
Intercept	–4.30*** (0.52)	–1.73* (0.73)	–2.58** (0.16)		–2.98*** (0.53)	2.96*** (0.40)		–4.97*** (0.60)		–4.79*** (0.60)
AIC:		2501.72			1937.55	7500.40		1792.85		1762.53

*N* = 2022 observations. Standard errors in parentheses. Degrees of freedom for mixed-models calculated using the Satterthwaite Approximation. Significance levels: *p* < .001***, *p* < .010**, *p* < .050*, *p* < .100^•^

As in Study 1, we then collapsed *D* and *D* + in order to focus on the decision to choose *S* + *.* Next, we conducted a series of mixed-model regressions controlling for: within-participant variance, whether or not participants indicated a preferred brand in the category, and demographic variables (degrees of freedom were calculated using the Satterthwaite approximation). Consistent with H1, a binomial logistic regression confirmed a linear relationship such that participants were less likely to choose *S* + as their expectation of demand due to COVID-19 increased (*b* = -0.65, *z*(1864) = -7.03, *p* < 0.0001, odds ratio = 1.92; Model 2-Table 5). A linear regression likewise confirmed that participants reported greater frustration on experiencing a stockout when their expectations of demand increase were lower (*M*_*high expectations*_ = 3.63, SE = 0.08; *M*_*unaffected expectations*_ = 3.93, SE = 0.06; *M*_*low expectations*_ = 4.29, SE = 0.10; *b* = -0.36, *t*(1578) = -7.92, *p* < 0.0001, *d* = 0.16; Model 3-Table 5). As in Study 1, this greater frustration predicted a higher likelihood of choosing the upgrade option *S* + (*b* = 0.55, *z*(1584) = 11.80, *p* < 0.0001, odds ratio = 1.74; Model 4-Table 5). Consistent with H2, a bootstrapped mediation analysis with 5000 iterations and standardized regression coefficients replicated the evidence for an indirect effect of participants’ expectations on upgrade behaviors through frustration (bias corrected 95% CI [-1.72, -0.81]; Model 5-Table 5). These results support the claim that consumers are more likely to show brand loyalty when they are less likely to expect a product shortage. As in Study 1, these results cannot be explained by resource scarcity or budget constraints arising from the pandemic.

## Study 3: Valentine’s Day gift choice

Thus far, we have used consumers’ prior experiences with product shortages (Study 1) and their expectations of demand increases during the COVID-19 pandemic (Study 2) to support the notion that consumers are more likely to accept a cost to stay brand loyal when a stockout is unexpected as compared to when it is expected. The main goal of Study 3 was to provide additional theoretical support for the proposed role of expectations in brand loyalty by manipulating and directly measuring participants’ expectations of product stockouts. A second goal of this study was to generalize the proposed effect beyond the pandemic context and thus demonstrate that it is robust to common shopping situations.

Study 3 was run in the month prior to Valentine’s Day, a holiday which entails large consumer demand for a somewhat narrow set of gift-related products. We manipulated consumers’ expectations of product shortages by having them simulate the experience of seeking a desired Valentine’s Day product with one day, one week, or one month remaining until the holiday itself. We predicted that being closer to (further from) the holiday would lead to greater (lower) expectations of stockouts, which would then lead consumers to feel less (more) frustrated and lead to correspondingly less (more) brand loyalty. Thus, the goal of this study was to test both H1 and H2 as part of this serial pathway.

### Method

This study used a 3-factor between-subjects design. U.S.-based respondents from Prolific (N = 202; *M*_age_ = 36.3, 72.9% female) were randomly assigned to conditions in which they read a scenario which asked them to simulate having one day, one week, or one month remaining until Valentine’s Day.

All participants completed an online survey in which they were asked to identify a product that they would genuinely be interested to buy as a gift for a significant other. They were provided URLs to dedicated Valentine’s Day ecommerce pages from Amazon, Target, and Walmart in order to find a real product. They then reported their selected product along with its brand and price. Next, participants completed a hypothetical scenario in which they attempted to acquire their desired product at a physical store with one day, one week, or one month remaining until Valentine’s Day (determined by condition). Because factors other than expectations may be impacted by the greater time pressure leading up to a deadline (for example, limited time could lead to greater desperation, which could lead to lower brand loyalty), we also measured and controlled for the extent to which participants believed it would be difficult to find and purchase their desired product by the deadline (1 = Not at all hard to 7 = Very hard).

Next, all participants were told that their desired product was out of stock and were asked to indicate both the degree to which they had expected such a stockout (1 = Not at all expected to 7 = Highly expected) and their frustration with the stockout (1 = Not at all frustrated to 7 = Extremely frustrated). They were then given a choice task in which they chose a substitute from between a more expensive product from the same brand as the original option (*S* +) and an equally priced alternative as the original option but from a different brand (*D*). Finally, we collected and controlled for age, gender, income, and whether or not the participants currently had a significant other (see materials in Appendix D).

### Results and discussion

We conducted a series of regressions to test our predictions (see Table 6). As predicted, the manipulation was successful such that less time remaining until Valentine’s Day linearly predicted higher expectations of a stockout (*b* = -0.69, *t*(195) = -4.69, *p* < 0.001 *R*^*2*^ = 0.11; Model 1-Table 6). Furthermore, less time remaining until Valentine’s Day also led to greater anticipated difficulty of finding the desired product (*b* = -0.99, *t*(195) = -6.64, *p* < 0.001, *R*^*2*^ = 0.1; Model 2-Table 6). As mentioned above, we controlled for this perceived difficulty in subsequent analyses in order to address otherwise unobserved negative impacts of the manipulation on participants’ behavior.

**Table 6:** Study 3 regression model results

Model number:	1	2	3	4	5	6
Model type:	Linear	Linear	Linear	Binomial logistic	Binomial logistic	Binomial logistic
DV:	Expectations	Difficulty	Frustration	Upgrade	Upgrade	Upgrade
Predictors						
Time pressure	–0.69*** (0.15)	–0.98*** (0.15)		0.40^•^ (0.22)		0.45* (0.22)
Expectations			-0.08** (0.03)			0.23* (0.10)
Frustration					0.20^•^ (0.11)	0.21 (0.23)
Difficulty			0.97*** (0.04)	0.20^*^ (0.10)	0.08 (0.09)	0.00 (0.25)
Age	–0.00 (0.01)	0.01 (0.01)	–0.01* (0.01)	–0.01 (0.01)	–0.01 (0.01)	–0.01 (0.01)
Gender (female)	–0.05 (0.27)	–0.12 (0.27)	0.02 (0.12)	–0.41 (0.35)	–0.44 (0.35)	–0.50 (0.36)
Income	0.00 (0.00)	0.00 (0.00)	0.00 (0.00)	0.00 (0.00)	0.00 (0.00)	0.00 (0.00)
Significant other	0.63* (0.28)	0.58 (0.28)	–0.20^•^ (0.12)	–0.66 (0.40)	–0.66 (0.41)	–0.75 (0.42)
Intercept	4.02*** (0.57)	4.10*** (0.57)	0.87*** (0.24)	–1.72^•^ (0.88)	–1.49^•^ (0.41)	–2.87** (1.03)
AIC:	793.85	798.53	452.93	248.87	248.47	245.81

*N* = 202 observations. Standard errors in parentheses. Significance levels: *p* < .001***, *p* < .010**, *p* < .050*, *p* < .100^•^

A logistic regression found a marginal effect of time remaining until Valentine’s Day on the upgrade decision such that more time until the deadline was associated with more brand loyalty, consistent with our claims (*b* = 0.40, *z*(194) = 1.78, *p* < 0.075, odds ratio = 1.49; Model 4-Table 6). We next examined our proposed mechanism by which more time until Valentine’s Day leads first to lower expectations of a stockout, then to greater frustration once that expectation is violated, and finally to greater brand loyalty. As expected, participants were indeed less likely to expect a stockout when there was more time until Valentine’s Day (Model 1-Table 6), and these lower expectations, in turn, led to greater frustration following the stockout (*b* = -0.08, *t*(194) = -2.66, *p* < 0.009, *R*^*2*^ = 0.81; Model 3-Table 6). Consistent with our predictions, this suggests that stockouts were less expected when the deadline did not loom, and hence led to greater frustration at the stockout. Also as expected, greater frustration predicted a higher likelihood of upgrading (*b* = 0.20, *z*(194) = 1.89, *p* < 0.059, odds ratio = 1.22; Model 5-Table 6). This serial pathway was tested with a bootstrapped serial mediation analysis using 5000 iterations, which showed that when there was more time until the deadline, this did indeed lead to more brand loyalty first through expectations (H1) and then frustration (H2; 95% CI[0.010, 0.367]; see Model 6-Table 6). In other words, the study shows that the proximity of Valentine’s Day impacted participants’ expectations of a stockout, and in turn, when stockouts were unexpected due to the date being further away, participants experienced greater frustration upon encountering a stockout which in turn led them to prefer an upgrade from the same brand as predicted.

## Study 4: Chocolate choice

Building on the hypothetical choice task of Study 3, this study makes use of a quasi-field design to extend the current findings in several significant ways. First, it entails an actual choice in which participants receive a product. Second, we directly manipulate whether participants are led to expect a product shortage instead of using indirect manipulations of expectation as in Studies 1–3. Third, we focus on expectations of stockouts arising from anticipation of reduced supplies rather than increased demand as in the previous studies, allowing us to generalize the findings. Finally, the studies thus far have examined brand loyalty through the lens of product upgrade decisions in which participants must trade off between price and brand loyalty. In this study, we generalize the findings by looking at a downgrade decision. Specifically, brand loyalty required participants to accept a smaller quantity of their desired product from their initially preferred brand versus selecting a larger quantity from a different brand. In addition to extending the examination beyond price-quality tradeoffs, using different quantities allowed us to keep the product constant and thereby remove any confounds that may have arisen due to beliefs about possible added value gained by upgrading.

### Method

This study used a 2 cell (Stockout: Expected vs. Not Expected) between-subjects design (pre-registered with AsPredicted, #88,117). Participants (N = 300) were pedestrians recruited in public on a U.S. university campus.

The study was run over the course of three days right after Valentine’s Day, and participants were told that in celebration of that holiday we were giving out free chocolate. They were given a choice between a 3-pack of Lindt chocolates and a 3-pack of Ferrero Rocher chocolates (see materials in Appendix E) and were asked to indicate which of these two options they wanted to receive. To manipulate expectation of shortage, half of the participants were told that supplies were limited and there was a chance they may not get their chosen chocolate (Stockout Expected condition), whereas the remaining participants were not provided any information about limited quantity (Stockout Not Expected condition).

All participants were then told to collect their chosen option at the Student Center, which was located away from the recruiting station. Upon reaching the Student Center, participants were greeted by a research assistant who guided them to one of two tables at different ends of the Student Center determined by which brand they had selected. Upon arriving at their designated table, participants were told that their chosen 3-pack option had run out, and they saw an empty bowl with the label of their chosen chocolate brand. To ensure that participants did not interpret this stockout as a signal that their chosen chocolate was particularly popular, they also saw an empty bowl labeled “Hershey’s.” Participants were told that while their preferred 3-pack was out-of-stock, they could choose a substitute of either a 3-pack from the brand which they had not selected previously or a 1-pack of chocolate from their originally chosen brand. Thus, participants who had initially chosen the 3-pack of Lindt (Ferrero Rocher) could choose between a 3-pack of Ferrero Rocher (Lindt) or a 1-pack of Lindt (Ferrero Rocher). Both the 3-packs and 1-packs were wrapped in transparent plastic bags (see Appendix E) to indicate that participants could not take more than one of a given pack. The research assistant then moved away to allow the participant to make their choice without any experimenter demand effects. At the conclusion of the study, we counted the number of 3-pack versus 1-pack chocolates chosen in each condition. We predicted greater choice of the 1-pack option when participants did not expect their chosen option to be out of stock (H1).

### Results and discussion

There was a significant preference among participants to stay with their initially chosen brand: 67.33% chose the 1-pack of chocolate from their initially chosen brand over the 3-pack from the other brand. However, in support of H1, more participants chose the 1-pack option from their initially chosen brand when the stockout was unexpected (73.33%) than when the stockout was expected (61.33%; *Χ*^*2*^(1) = 4.91; *p* < 0.028; odds ratio = 0.58). Thus, participants were more willing to trade off on quantity to remain brand loyal when they did not (vs. did) expect stockouts. Notably, this effect was found using real choices in a field setting by directly manipulating the expectations of the stockout.

## Study 5: Bike choice

While we have thus far established the robustness of the effect of expectations and frustration on brand loyalty, we have yet to directly test the underlying process. Therefore, the first objective of Study 5 is to provide direct evidence for our proposed affect-based mechanism which states that unexpected stockouts lead to greater negative affect than expected stockouts and thus consumers are more likely to choose a same-brand substitute in order to resolve this negative affective state. In other words, we have proposed that brand loyalty arises largely because participants seek affect-rich products after unexpected stockouts, and the brand is a more affect-rich attribute than non-brand attributes like price and quantity. However, a number of alternative accounts exist to explain our pattern of results: for example, it is possible that consumers pick a substitute from the same brand as a heuristic, e.g., to simplify their choice, avoid deliberation, or minimizes time investment. Therefore, the second objective of this study is to rule out such alternative explanations that posit a simple or strict preference for the same brand following an unexpected stockout.

A third objective of this study is to test H3, thereby establishing a boundary on the proposed effect to provide both theoretical support as well as greater managerial insight into when we may or may not expect to see brand loyalty in the face of stockouts. To achieve these objectives, Study 5 looks at tradeoffs between the brand and non-brand attributes other than price and quantity, to which our investigation thus far has been limited. Brand-price and brand-quantity tradeoffs are important and extensible contexts wherein brand is likely the more affect-rich attribute under consideration, but there can be contexts in which the affective value of the brand is matched or even overshadowed by that of another attribute (e.g., hedonic characteristics of a product or a charitable cause associated with it). In such contexts, our affect-based account would not predict brand loyalty after an unexpected stockout as consumes should instead be drawn to the non-brand attributes that deliver higher affective value than the brand.

### Method

This study used a 3 cell (Alternative Brand Option: No Upgrade, Affective Upgrade, Non-affective Upgrade) between-subjects design (pre-registered with AsPredicted, **#**107,268). Participants (N = 733) were undergraduate students at a U.S. university who completed the study as part of a larger lab session to meet course requirements. Participants were randomly assigned to a condition.

All participants were presented a scenario where they imagined that they were shopping for a bicycle. They were all then shown a bicycle with certain features, including the brand (see stimuli in Appendix F), and were told that they had identified it as their ideal choice after some research. Next, participants were asked to imagine that they traveled to a store and went to the bikes section to find their desired bike. At this point, they were asked to indicate their expectations that the specific bike they were looking for would be in-stock (1 = Definitely in-stock to 7 = Definitely not in-stock). Participants were subsequently told to imagine that the bike they wanted was out-of-stock.

We next showed participants two substitute bikes from which they could choose. One option was the same for all participants: it was from the same brand as the stocked-out bike, had very similar features to the original option, but was higher in price. The alternative option was always from a different brand and its features were determined by condition: in the No Upgrade condition the alternative bike had the same features and price as the stocked-out bike; in the Affective Upgrade condition the alternative offered a stylish frame and handlebars and a customizable color/paint job at the same higher price as the same-brand option; and in the Non-affective Upgrade condition the alternative came with precision brakes and shock absorbers at the same higher price as the same-brand option. The stylish frame and handlebars and the customizable color/paint job were used as affective features while the precision brakes and shock absorbers were used as non-affective features based on a separate test (N = 141), which confirmed that participants viewed brand as having more affective value than brakes and shock absorbers but less affective value than stylish frame/handlebars and customizable color/paint (pretest results in Web Appendix 1). The brands used in the study were Schwinn and GIANT, two popular brands in the United States. We randomized which was the original brand and which was the different brand to avoid stimulus effects. After indicating their choice, participants completed a manipulation check where they rated the different bike attributes including the brand, stylish frame and handlebars, customizable color/paint, precision brakes, and shock absorbers on two items: “In general, I would prioritize a bike with this attribute for its…” (1 = practical value to 7 = Emotional value) and “I would determine the value of this attribute with…” (1 = careful reasoning to 7 = personal feelings).

Our prior findings predict that when expectations of the stockout are low, participants should be more likely to choose the same-brand upgrade over the No Upgrade alternative-brand substitute because the brand delivers more affective value. Alternative accounts, such as heuristics or reactance, predict this patten to persist regardless of the affective value of the option from the alternative brand. However, our account predicts that if an alternative offers more affective features, participants should then be more likely to switch to that alternative in order to achieve a more affectively rewarding outcome. Thus, we would predict that when the alternative brand option is an Affective Upgrade, this will moderate the effect of an unexpected stockout on participants’ preference for the same-brand option. To demonstrate that this is not driven by a mere preference for upgrades, we included the Non-affective Upgrade alternative, and predict that the pattern of results in that condition will be the same as in the No Upgrade condition and as observed in our prior studies.

### Results and discussion

The manipulation checks confirmed our claim that the brand’s affective value (*M* = 2.75, SE = 0.09; *α* = 0.77) was seen as less than the aggregate affective value of the stylish frame and handlebars as well as the customizable color/paint attributes of the Affective Upgrade (*M* = 4.65, SE = 0.05; *α* = 0.79; *t*(1464) = -25.63, *p* < 0.0001, *d* = 1.34) but greater than the aggregate affective value of the precision brakes and shock absorbers attributes of the Non-affective Upgrade (*M* = 1.63, SE = 0.04; *α* = 0.86; *t*(1464) = 17.67, *p* < 0.0001, *d* = 0.92).

**Fig. 1:**
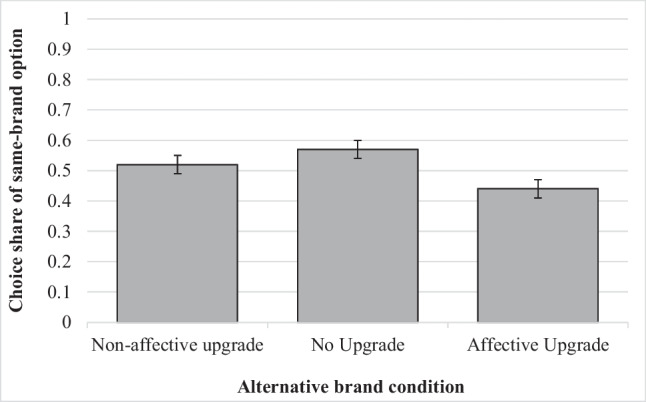
Choice shares of the same-brand option in Study 5

Participants’ preference for the same-brand substitute was predicted with a binomial logistic regression on the basis of condition and expectations while controlling for the brand used in the stimuli (there were no effects of this control, so it is not discussed further). As we predicted divergent effects for the Affective Upgrade condition relative to the No Upgrade and Non-affective Upgrade conditions, the conditions were coded to compare No Upgrade to Affective Upgrade and No Upgrade to Non-affective Upgrade (in essence treating No Upgrade as the control condition). In line with our previous findings, we found a significant main effect of expectations such that higher expectations of a stockout led to lower choice share of the same-brand option (*B* = -1.69, *z*(732) = -8.52, *p* < 0.0001, odds ratio = 0.18). Also as predicted, we observed no difference between No Upgrade and Non-affective Upgrade conditions (*B* = 1.44, *z*(732) = 1.14, *p* = 0.253, odds ratio = 4.22) but a significant decrease in the choice of the same-brand option between No Upgrade and Affective Upgrade conditions (*B* = -9.16, *z*(732) = -10.31, *p* < 0.0001, odds ratio = 0.00), consistent with the prediction that participants presented with an affectively attractive alternative to the same-brand option will switch to it in pursuit of greater affective value (see Fig. 1). We further observed a significant interaction between the Affective Upgrade comparison and expectations (*B* = 2.43, *z*(732) = 10.25, *p* < 0.0001, odds ratio = 11.33) and no such interaction with the Non-affective Upgrade comparison (*B* = -0.36, *z*(732) = -1.11, *p* = 0.269, odds ratio = 0.70). Decomposing the interaction, we observed that although the negative relationship between expectations and choice of the same-brand substitute was observable within the No Upgrade (*B* = -1.68, *z*(253) = -8.48, *p* < 0.0001, odds ratio = 0.19) and Non-affective Upgrade conditions (*B* = -2.05, *z*(239) = -8.03, *p* < 0.0001, odds ratio = 0.13), this effect was reversed in the Affective Upgrade condition (*B* = 0.74, *z*(238) = 5.74, *p* < 0.0001, odds ratio = 2.09). Thus, participants facing an unexpected stockout, and who would thus generally prefer the same-brand option, instead opted for an affectively superior alternative when presented with one.

This study provides support for H3 and demonstrates that consumers faced with an unexpected stockout tend to prefer substitutes with the greatest affective value, whether that is an option with the same brand or an option with affectively superior non-brand features. This result supports our affect-based account for explaining the phenomena observed in prior studies as it is consistent with the claim that an unexpected stockout leads consumers to choose a same-brand substitute when the brand provides greater affective value than non-brand attributes. These data thus show an important boundary condition of our proposed effect: when alternative brands offer higher affective value, we will observe reduced brand loyalty in the face of unexpected stockouts. More broadly, the data suggest that we should generally expect to see consumers preferring options with more affect-rich attributes when faced with an unexpected (vs. expected) stockout. Thus, Study 5 recommends that managers might benefit from efforts that magnify or supplement the perceived affective value of their product when aiming to secure brand loyalty or to capitalize on an unexpected stockout. Lastly, the results of this study rule out any alternative accounts for our proposed effect which would predict a strict or simple preference for a same-brand substitute following a stockout, such as a heuristic to always choose a favored brand.

## General discussion

Demand increases, supply shortages, limited product runs, capacity constraints, and global trade disruptions can all result in product shortages and stockout. Firms may even create product shortages intentionally by holding supplies artificially low or by generating perceptions of scarcity through marketing communications. Therefore, it is critical for managers to accurately anticipate how consumers may respond to such shortages. In the current article, we focus on the important question of whether consumers are more likely to stay loyal to a brand when faced with product stockouts or are more likely to switch to a different brand in pursuit of a better deal. We propose and demonstrate that consumers are more likely to remain brand loyal when they do not expect product shortages, even if that means buying options that are more expensive or options that offer a smaller quantity (H1). We demonstrate that this pattern of behavior arises because consumers feel greater frustration upon encountering an unexpected stockout (H2). We reason that this greater negative affect leads consumers to choose a substitute that provides greater affective value in order to ameliorate their negative feelings. As brand is a relatively affect-rich product attribute compared to many non-brand attributes such as price and quantity, consumers facing an unexpected stockout are more likely to choose a substitute from the same brand and thus demonstrate brand loyalty. However, when given the ability to satisfy this preference for affective value by selecting other, more affect-rich products, we observe that they do so (H3).

### Theoretical contributions

Our findings make important theoretical contributions to a number of literatures. We contribute to prior research on substitution by highlighting the role of consumers’ expectations, and the role of attributes’ affective value, in substitution decisions. We note that while prior research has identified certain specific negative emotional reactions that lead to apparent preferences for similar substitutes, namely the experiences of reactance or jilting (i.e., Clee & Wicklund, 1980; Fitzsimons, 2000; Litt et al., 2010), these explanations are specific to the emotions themselves. In other words, they rely more on consumers’ appraisals of those emotions—the cognitive or behavioral imperatives that accompany these specific emotional states (Smith & Ellsworth, 1985)—rather than examining the consequences of the negative affect more broadly. Thus, our approach offers a more generalizable and parsimonious explanation for consumers’ decisions when faced by a stockout. In further support of our approach, we note that we do not observe anything that could be considered a preference for similarity in Study 5 when participants were given the option to choose an alternative with greater affective value. This is inconsistent with prior models based on reactance or jilting, which suggest that a preference for similarity should dominate. Thus, our research establishes when an apparent preference for similarity is more accurately characterized as a preference for greater affective value that happens to be offered by similar substitutes.

We likewise add to research examining product and attribute preferences under scarcity. Much of this work has emphasized consumers’ motivations (Brannon & Brock, 2001; Inman et al., 1997; Suri et al., 2007; Wu & Lee, 2016) and perceptions (Balachander & Stock, 2009) rather than their expectations or the affective value of product attributes. We expand on this research by digging deeper than a general preference for similarity under scarcity (e.g., Arens & Hamilton, 2016; Huh et al., 2016) to shed light on when such a preference may arise and on what dimensions consumers may seek similarity. In a similar fashion, we expand on extant research on expectations, which provided a basis for our observations that lower expectations of stockouts will generate more intense frustration (e.g., Wilson et al., 1989), but likewise does not provide insights into the potential preference for brand loyalty. We thus provide a synthesis and extension of the literatures on scarcity, expectations, and affect-driven consumption to generate a novel prediction suggesting that consumers are more likely to prefer a similar brand when the stockout is unexpected and when the brand provides higher affective value relative to other non-brand attributes. In doing so, we also advance the research on branding by proposing expectations of a stockout as a novel antecedent to brand loyalty. Our work thus also adds to research on consumer choice and the preference construction process (e.g., Bettman et al., 1998; Khan et al., 2011) by shedding light on how consumers make tradeoffs between brand and the non-brand attributes. Finally, our research contributes to the growing study of global trade disruptions, such as those caused by the pandemic (e.g., Das et al., 2021; Goldsmith & Lee, 2022), that can create or exacerbate product shortages.

### Managerial contributions

Our findings provide important insight for firms that may face reputational and financial hazards stemming from stockouts and shortages. Our research suggests that it is prudent to both consider and manage consumer expectations when guiding commercial strategies about when to focus on price incentives versus when to leverage brand loyalty to upsell. The results are particularly relevant for firms with products for which consumers may not anticipate a stockout (for example, in categories that are believed to be unrelated to a market shock, such as durable products during the COIVD-19 pandemic) and for firms with deep product lines that present a ready opportunity to promote upgrade substitutes. It should also be noted that competitive strategies must be different for brands that compete in categories involving more (vs. fewer) affect-rich products and attributes. Our general takeaway is relevant to market leaders with strong brand reputations, but also more broadly in categories or industries in which we would expect that the brand will have relatively high affective value relative to other important non-brand attributes. This would include utilitarian products as well as categories in which price and/or quantity are the key levers of differentiation. Meanwhile, in hedonic or symbolic product categories in which competing alternatives may have very high affective value stemming from attributes other than brand, we would not expect to observe greater brand loyalty following an unexpected shortage. We might, therefore, also advise that managers seek to magnify the perceived affect-richness of their products when stockouts are possible to retain or to capture alternative-seeking consumer, especially in the case of companies with weaker brand reputations. Furthermore, managers may expect to benefit from efforts intended to enhance the perceived affective value of their brand, which will allow them to continue to leverage shortages to promote brand loyalty by appealing to consumers grappling with unexpected stockouts.

Our findings are illuminating given that our survey of managers shows that most did not seem to have the right intuition about how expectations of stockouts impact brand loyalty. Interestingly, the more experience that managers had, the more erroneous their predictions seem to be. Our results thus also have implication for transparency practices and research. The last two decades have seen a heightened push towards transparency in corporate behavior, operations, and communication. Some argue that transparency is central to business success, and that the best approach is to always be more transparent (e.g., Tapscott & Ticoll, 2003). This sentiment was also voiced by managers in our survey (e.g., “Transparency is always the most important”, and “I believe customers would appreciate the honesty and candor”). Our research suggests that managers should engage in more thoughtful consideration of the role of their customers’ expectations as they make critical decisions about information disclosure.

### Future directions and limitations

Our theoretical framework suggests several potential moderators and boundaries of the effect of stockouts on brand loyalty that invite future examination. For example, consumers who feel emotions with greater intensity (vs. those who experience emotions with lesser intensity; Larsen & Diener, 1987) may demonstrate greater brand loyalty in the face of unexpected stockouts rather than look for better deals. Similarly, situational contexts (e.g., global or national calamities) may heighten affective responses and the extent of the observed effects. Whether a consumer interprets a stockout as temporary versus permanent may also impact brand loyalty, as could the relative cost (in time or money) of finding the originally desired product elsewhere. In the current research, we conceived negative affect as frustration directed at the stockout itself, however if such feelings were instead directed at the company for failing to stock the product, this could potentially moderate the effect. Finally, past research suggests that consumers of low socioeconomic status tend to experience less reactance when denied a desired product (Snibbe & Markus, 2005; Thompson et al., 2020). While we did not find any moderating effect of income in our data, future research can examine in more detail when consumers’ own resource and socioeconomic limitations (Cannon et al., 2018; Sharma & Alter, 2012) may or may not play a role in brand loyalty.

Our research is not without limitations, which in turn present several directions for further inquiry. First and foremost, we have identified a key boundary to our proposed effect, which is that consumers may not show brand loyalty when affect-rich alternatives exist. This boundary suggests that a complete view of how affect informs decisions between substitutes warrants a rigorous measurement of the affective value of different attributes, and a deeper understanding of how this affective value may shift depending on different contexts, such as the types of products, types of sellers, or types of consumers. For instance, while we considered contexts in which the attributes of price and quantity were likely to be affect-poor, there may be situations in which one or both of these attributes are endowed by emotional value. For example, price may carry high affective value if there is an unexpectedly large discount or if the price is unexpectedly high or seen as unfair (Campbell, 2007). Our current results should be understood with these boundaries in mind, and future explorations should investigate factors that may influence the affective value of non-brand attributes and of the brand relative to those other attributes. Furthermore, future work may explore how effective brand versus other affect-rich attributes are in actually mitigating the negative affect caused by a stockout.

Future research may also meaningfully expand upon our work by looking at the antecedents of consumer stockout expectations. While our exploration of the phenomenon was limited to the ways in which consumers’ own experiences, their predictions of demand increases, and their exposure to messaging about shortages contribute to perceptions of stockout likelihoods, many other factors could influence these expectations further. One such potential future avenue would be to look at stable or chronic antecedents of stockout expectations: for example, consumers may form such expectations differently depending on whether the product is inherently seen as a necessity versus a luxury, is of local versus foreign origin, or is from a durable versus consumable product category. The issue of durable versus consumable products is one that can be explored in the data from Studies 1 and 2 given the range of categories evaluated. We conducted such an initial analysis (see Web Appendix 2) and found that participants were more likely to remain brand loyal when faced with stockouts of durable (vs. consumable) products. We interpret these results as being consistent with our prediction that brand loyalty is driven by expectations as participants were less likely to have encountered stockouts of durables (Study 1) and were less likely to assume that COVID-19 would impact demand for them (Study 2). However, future research might explore inherent differences in how stockouts impact expectations and brand loyalty as a function of stable characteristics of product categories or markets. Another fruitful direction for future research would be to extend the investigation of the phenomenon beyond stockouts and into other domains of scarcity (e.g., limited time availability) as different types of scarcity can have distinct impacts on consumer behavior (e.g., Aggarwal et al., 2011; Kristofferson et al., 2017).

Lastly, our results offer an opportunity to apply and further explore our affect-based mechanism beyond the substantive context of brand loyalty. For example, future work may investigate whether a preference for affect-richness is robust across contexts in which a consumer seeks a substitute following a failure to acquire a desired product. Our framework would predict that if consumers face a stockout that is unexpected rather than anticipated, they are more likely to pick affect-rich alternatives, such as options which are more hedonic, indulgent, aesthetically-pleasing, etc. (Khan et al., 2005). More broadly, future research could interrogate orthogonal sources of affective value in order to better understand the processes that underly substitution decisions, such as by looking at the intensity or arousal of affect associated with an alternative rather than simply the positive valence (e.g., Wilson et al., 1989). A separate means by which future research might extend the findings beyond the context of brand loyalty would be to explore the extent to which a preference for affect-richness also underlies different or more general forms of preferences for similarity. For example, products, attributes, or stimuli in general that are familiar to a consumer are known to carry more affective value (Zajonc & Markus, 1982), and therefore we may predict that, all else equal, more similar substitutes will be seen as more affect-rich than less similar substitutes, thus providing an alternative understanding of the preference for similarity observed in prior literature.

### Supplementary Information

Below is the link to the electronic supplementary material.

Supplementary file1 (DOCX 1142 kb)

## Appendix A: Managers’ intuition about stockouts and brand loyalty

In this study, we aimed to highlight the importance of our findings by demonstrating that firms do not have a strong understanding of how expectations of stockouts impact brand loyalty. To illustrate this point, we presented managers with a scenario like the one in this article’s opening example and invited them to advise on what a company anticipating a stockout should do to ensure brand loyalty.

### Method

We distributed a survey to executives and managers who were alumni of a U.S. business school via an email which invited them to provide their input in a business study (pre-registered with AsPredicted, #89553). We chose this population to ensure that participants had training and experience in business disciplines. The study was run for a two-week period. The survey link was opened by 1,145 participants, of which 876 provided a response to the main question and 735 completed the full survey.

Participants read the scenario below in which they were asked to advise a large company experiencing a supply chain problem that would result in possible stockouts for their customers. After providing their recommendation, participants briefly explained their reasoning. We predicted that managers and executives would not have the understanding that consumers who expect to encounter a product stockout (vs. those who do not expect it) will be less likely to choose a substitute from the same brand rather than from a competitor. Lastly, all participants indicated how many years of experience they had and the industry in which they currently worked.

**Table:**

Suppose you are advising a large company. This company has identified that, due to supply-chain disruptions, they will likely not be able to meet demand through their retail channels in a specific product category. The company has already implemented a plan to solve the supply chain problem, but in the meantime, some of their customers might encounter stock-outs of their products in the coming weeks. Leadership's primary concern is losing market share as customers may switch to competitors. The company has multiple products available in this category, but the product that will be out-of-stock is their lowest price option. Leadership wants to maximize the likelihood that their customers choose to buy a more expensive alternative product from them rather than defecting to a competitor’s product. The specific question on which you are asked to advise is **whether it is more prudent to make public the information about the potential product shortage or to suppress this information. In other words, whether it makes more sense to advise customers about the possibility that they may encounter a stock-out, or to let them be surprised if they encounter such a stock-out.** What would you advise the company to do to increase the likelihood that they don’t lose customers to competitors?	
Make the information about the potential stock-out public	Do **not** make the information about the potential stock-out public

### Results and discussion

We report results from those who completed the survey (N = 735; *M*_*experience*_ = 25.4 years, *SD* = 13.6 years). Including incomplete responses in the analysis does not change the results.

Consistent with our prediction, 80.30% of the respondents recommended informing customers of potential stockouts, whereas only 19.70% recommended not making the information public (binomial test: 95% CI[0.77, 0.83], *p* < .0001). This shows that managers’ intuitions are opposite to how expectations of stockouts actually influence brand loyalty. Additionally, a binomial logistic regression examined whether respondents’ predictions improve with their number of years’ experience and found the opposite to be true (*b* = -0.02, *z*(733) = -2.46, *p* < .014, odds ratio = 0.98).

**Replications** We further conducted the same survey using a novice population that is less likely to have business training and experience. Specifically, MTurk workers (*N* = 212) provided their recommendation after reading the same scenario. The results were similar to the manger population: 89.15% of the respondents recommended informing customers of the potential stockouts, while only 10.85% recommended not making the information public (binomial test: 95% CI[0.84, 0.93], *p* < .0001). Thus, expert managers and executives appear to be no better than novices at predicting the effect of stockout expectations on brand loyalty.

We conducted yet another variant of the survey to address a potential concern that the scenario may have failed to sufficiently focus respondents on how their recommendation would impact the expectations of stockouts. MTurk workers (*N* = 277) saw the following scenario and indicated which of two consumers was more likely to choose an upgraded product from a desired brand rather than to switch to a different brand when faced with a stockout

**Table:**

Suppose there are two customers, John and Michael. Both are similar in their financial, economic, and social backgrounds. Both John and Michael separately arrive at a retail store seeking the same product from the same brand. Both John and Michael discover that their desired product is out-of-stock. • John expected that there was a possibility that this could be the case. • Michael had no expectation that the product could be out-of-stock. While their desired product is out-of-stock, the store has two potential substitute products that the customers may choose from:	
Product A	Product B
This is a more expensive product from the same brand which they originally desired	This product is of equal price to the originally desired product but from a different brand
Who, in your opinion, is more likely to choose Product B, i.e., the expensive product from the originally desired brand? • John is more likely to choose Product B than Michael • Michael is more likely to choose Product B than John • John and Michael are equally likely to choose Product B	

Consistent with the previous two versions of this question, 45.85% of participants thought that John (who expected the stockout) was more likely to upgrade and stay brand loyal, while only 31.05% thought that Michael was more likely to stay brand loyal and 23.10% thought they were equally likely to stay brand loyal (*Χ*^*2*^(2) = 22.14, *p* < .0001). Once more, participants failed to forecast the role of expectations correctly.

## Appendix B: Studies summary

**Table:**

	**Key Finding**	**Sample size & Population**	**Design**
**Introductory surveys**	Neither managers nor novice respondents accurately predicted that greater expectation of a stockout will lead to less brand loyalty.	*N* = 735 (Business school alumni) *Replications:* *N* = 212 (MTurk) *N* = 277 (MTurk)	Survey DV: Choice between whether expected (vs. unexpected) shortages will lead to greater brand loyalty
**Study 1**	Participants who have experienced stockouts of a desired product were less likely to be frustrated with a stockout, and this led them to be less likely to remain loyal to a brand from which they intended to buy.	*N* = 303 (MTurk) *M*_age_ = 37.2 39.9% female	Survey IV: Experience of shortages by product category DV: Choice of an upgrade option to remain brand loyal
**Study 2: Main**	Participants who expected greater demand for a desired product (as a function of relevance during the COVID-19 pandemic) were less likely to be frustrated with a stockout, and this led them to be less likely to remain loyal to a brand from which they intended to buy.	N = 674 (MTurk) *M*_age_ = 36.0 49.7% female	Survey IV: Product category (varying by expectations about demand and stockouts) DV: Choice of an upgrade option to remain brand loyal
**Study 2 pretest: Demand expectations**	Participants held systematic beliefs about the product categories for which COVID-19 increased demand, had no effect on demand, or reduced demand.	*N* = 102 (MTurk) *M*_age_ = 39.3 46.8% female	Survey IV: Product category DV: Level of agreement (out of 100) that COVID-19 impacted demand for a given category
**Study 2 pretest: Demand-shortage link**	Participants held the belief that increased consumer demand during COVID-19 led to product stockouts.	*N* = 104 (MTurk) *M*_*age*_ = 39.1 40.4% female	Survey DV: Expectations about stockouts given increased demand
**Study 3**	Participants expected a greater likelihood of a stockout as demand for a product increased (as a function of a looming holiday), and as a result were less likely to be frustrated with a stockout, which made them less brand loyal.	*N* = 202 (Prolific) *M*_age_ = 36.3 72.9% female	Survey IV: Time until Valentine’s Day: 1 day vs. 1 week vs. 1 month DV: Choice of an upgrade option to remain brand loyal
**Study 4**	Participants who expected (vs. not expect) a stockout were less likely to remain loyal to a brand which they initially preferred.	*N* = 300 (University students)	Field experiment IV: Stockout Expected vs. Not Expected DV: Choice of a downgrade option to remain brand loyal
**Study 5**	Participants with lower expectations of a stockout were more likely to prefer the more affect-rich substitute, whether it was the same brand or an affective upgrade.	*N* = 733 (University students)	Survey IV: Expectations of a stockouts; Brand substitute: not an upgrade vs. an affective upgrade vs. a non-affective upgrade DV: Choice of a brand loyal upgrade option
**Study 5 pretest: Attribute affective value**	Brand as an attribute was rated as having greater affective value than practical product attributes but less than explicitly affective attributes such as aesthetics.	*N* = 141 (University students)	Survey: IV: Affective value ratings of a selection of bicycle attributes

## Appendix C: Materials for Studies 1 and 2

### Brand preference and stockout experience questions (toilet paper category).

Note that each question appeared separately, and that the “What is your preferred brand of toilet paper?” item was only shown to participants who indicated “Yes” on the first question.

### Stockout scenario (toilet paper category)

The following was given if a participant indicated no brand preference. Participants who indicated a preferred brand saw the name of that brand instead of the word “usual” below.

## Appendix D: Materials for Study 3

### Stockout questions (1 week condition).

Participants are asked to identify the name of a retailer where they would look for the product. Note that participants saw the name of the product they entered in place of [product] below.

They were then shown the following. Note that participants saw the name of the store they entered above in place of [retailer name] below.

Participates then made their upgrade choice. Note that participants saw the brand name and price that they previously entered in place of [brand] and ($0.00) below respectively.

## Appendix E: Materials for Study 4

**Table:**

Stockout Expected Condition	Stockout Not Expected Condition
	

## Appendix F: Materials for Study 5

These materials were presented to participants as pen-and-paper activities to be completed one page at a time. Six versions of the survey were printed and randomly distributed to students: 3 (Alternative Brand Option: No Upgrade vs. Affective Upgrade vs. Non-affective Upgrade) x 2 (Randomized initial brand: GIANT vs. Schwinn).

**Page 1 (same for all Alternative Brand Options, shown with GIANT initial brand)**

**Suppose you are shopping for a bicycle.**

**After a bit of research, you identify the following bike as your ideal choice.**

**Please examine this bike** **carefully****:**

**Table:**

	• **Brand:** GIANT^TM^ • **Price:** $329.99 • Aluminum frame • Standard brakes • Polymer seat • Standard tires

**Page 2 (same for all versions)**

**Please imagine yourself leaving home and traveling to a local store that sells bikes…**

**Now imagine yourself entering the store and going to the section where the bikes are sold…**

What would be your expectation that the specific bicycle that you want would be in stock? Circle your response.

**Table:**

Definitely in-stock						Definitely **not** in-stock
**1**	**2**	**3**	**4**	**5**	**6**	**7**

**Page 3 (No Upgrade Condition, GIANT initial brand)**

**As you get to the bikes section, you discover that the store is** **sold out** **of the specific Schwinn bike you were looking for.**

**However, the store still has two other bikes you could purchase.**

**Suppose that you decide that you will choose one of these two alternative options.** **Circle the bike you would buy****:**

**Table:**

	• **Brand:** GIANT^TM^ • **Price:** $400.00 • Aluminum frame+ • Standard brakes • Polymer seat • Standard tires
	• **Brand:** Schwinn^TM^ • **Price:** $329.99 • Aluminum • Standard brakes • Polymer seat
**Page 3 alternate alternative (Affective Upgrade Condition, GIANT initial brand)**	
	• **Brand:** Schwinn^TM^ • **Price:** $400.00 • Stylish frame and handlebars • Customizable paintjob • Standard brakes • Polymer seat
**Page 3 alternate alternative (Non-Affective Upgrade Condition, GIANT initial brand)**	
	• **Brand:** Schwinn^TM^ • **Price:** $400.00 • Aluminum frame • Precision brakes • Shock absorbers • Polymer seat

**Page 4 (same for all versions)**

**Tell us what you care about for each of these bike attributes:**

**Table:**

The bicycle brand						
In general, I would prioritize a bike with this attribute for its...						
**Practical value**						**Emotional value**
**1**	**2**	**3**	**4**	**5**	**6**	**7**
I would determine the value of this attribute with…						
**Careful reasoning**						**Personal feelings**
**1**	**2**	**3**	**4**	**5**	**6**	**7**
Stylish frame and handlebars						
In general, I would prioritize a bike with this attribute for its...						
**Practical value**						**Emotional value**
**1**	**2**	**3**	**4**	**5**	**6**	**7**
I would determine the value of this attribute with…						
**Careful reasoning**						**Personal feelings**
**1**	**2**	**3**	**4**	**5**	**6**	**7**
Customizable color or paintjob						
In general, I would prioritize a bike with this attribute for its...						
**Practical value**						**Emotional value**
**1**	**2**	**3**	**4**	**5**	**6**	**7**
I would determine the value of this attribute with…						
**Careful reasoning**						**Personal feelings**
**1**	**2**	**3**	**4**	**5**	**6**	**7**
Precision brakes						
In general, I would prioritize a bike with this attribute for its...						
**Practical value**						**Emotional value**
**1**	**2**	**3**	**4**	**5**	**6**	**7**
I would determine the value of this attribute with…						
**Careful reasoning**						**Personal feelings**
**1**	**2**	**3**	**4**	**5**	**6**	**7**
Shock absorbers						
In general, I would prioritize a bike with this attribute for its...						
**Practical value**						**Emotional value**
**1**	**2**	**3**	**4**	**5**	**6**	**7**
I would determine the value of this attribute with…						
**Careful reasoning**						**Personal feelings**
**1**	**2**	**3**	**4**	**5**	**6**	**7**

## References

[CR1] Aaker DA (1991). Managing brand equity: Capitalizing on the value of a brand name.

[CR2] Aggarwal P (2004). The effects of brand relationship norms on consumer attitudes and behavior. Journal of Consumer Research.

[CR3] Aggarwal P, Jun SY, Huh JH (2011). Scarcity messages. Journal of Advertising.

[CR4] Alba JW, Williams EF (2013). Pleasure principles: A Review of research on hedonic consumption. Journal of Consumer Psychology.

[CR5] Andrade EB (2005). Behavioral consequences of affect: Combining evaluative and regulatory mechanisms. Journal of Consumer Research.

[CR6] Arens ZG, Hamilton RW (2016). Why focusing on the similarity of substitutes leaves a lot to be desired. Journal of Consumer Research.

[CR7] Balachander S, Stock A (2009). Limited edition products: When and when not to offer them. Marketing Science.

[CR8] Batra R, Ahtola OT (1991). Measuring the hedonic and utilitarian sources of consumer attitudes. Marketing Letters.

[CR9] Becdach C, Brown B, Halbardier F, Henstorf B, Murphy R (2020). Rapidly forecasting demand and adapting commercial plans in a pandemic.

[CR10] Berger J, Heath C (2007). Where consumers diverge from others: Identity signaling and product domains. Journal of Consumer Research.

[CR11] Bettman JR, Luce MF, Payne JW (1998). Constructive consumer choice processes. Journal of Consumer Research.

[CR12] Borkovsky RN, Goldfarb A, Haviv AM, Moorthy S (2017). Measuring and understanding brand value in a dynamic model of brand management. Marketing Science.

[CR13] Brannon LA, Brock TC (2001). Scarcity claims elicit extreme responding to persuasive messages: Role of cognitive elaboration. Personality and Social Psychology Bulletin.

[CR14] Braun OL, Wicklund RA (1989). Psychological antecedents of conspicuous consumption. Journal of Economic Psychology.

[CR15] Campbell MC (2007). "Says who?!" How the source of price information and affect influence perceived price (un)fairness. Journal of Marketing Research.

[CR16] Cannon C, Goldsmith K, Roux C (2018). A self-regulatory model of resource scarcity. Journal of Consumer Psychology.

[CR17] Cavallo A., & Kryvtsov O. (2021). What can stockouts tell us about inflation? *Evidence from online micro data, NBER Working Paper 29209*.

[CR18] Chernev A, Hamilton R, Gal D (2011). Competing for consumer identity: Limits to self-expression and the perils of lifestyle branding. Journal of Marketing.

[CR19] Cialdini RB, Darby BL, Vincent JE (1973). Transgression and altruism: A case for hedonism. Journal of Experimental Social Psychology.

[CR20] Clee MA, Wicklund RA (1980). Consumer behavior and psychological reactance. Journal of Consumer Research.

[CR21] Das G, Jain SP, Maheswaran D, Slotegraaf RJ, Srinivasan R (2021). Pandemics and marketing: Insights, impacts, and research opportunities. Journal of the Academy of Marketing Science.

[CR22] Day GS (1969). A two-dimensional concept to brand loyalty. Journal of Advertising Research.

[CR23] Fitzsimons G (2000). Consumer response to stockouts. Journal of Consumer Research.

[CR24] Folkes VS (1988). The availability heuristic and perceived risk. Journal of Consumer Research.

[CR25] Fournier S (1998). Consumers and their brands: Developing relationship theory in consumer research. Journal of Consumer Research.

[CR26] Friedman EMS, Toubia O (2022). Pricing fairness in a pandemic: Navigating unintended changes to value or cost. Journal of the Association for Consumer Research.

[CR27] Geers AL, Lassiter GD (1999). Affective expectations and information gain: Evidence for assimilation and contrast effects in affective experience. Journal of Experimental Social Psychology.

[CR28] Gelbrich K (2010). Anger, frustration, and helplessness after service failure: Coping strategies and effective informational support. Journal of the Academy of Marketing Science.

[CR29] Gierl H, Huettl V (2010). Are scarce products always more attractive? The interaction of different types of scarcity signals with products' suitability for conspicuous consumption. International Journal of Research in Marketing.

[CR30] Goldsmith, K. & Lee, A. Y. (2022). Flash COVID-19 Research Issue: Insights on COVID-19 Outbreak and Related Topic. *Journal of the Association for Consumer Research, 6*.

[CR31] Grisaffe DB, Nguyen HP (2011). Antecedents of emotional attachments to brands. Journal of Business Research.

[CR32] Hamilton RW, Thompson DV, Arens ZG, Blanchard SJ, Haubl G, Kannan PK, Khan U, Lehmann DR, Meloy M, Roese NJ, Thomas M (2014). Consumer substitution decisions: An integrative framework. Marketing Letters.

[CR33] Hamilton RW, Thompson D, Bone S, Chaplin LN, Griskevicius V, Goldsmith K, Hill R, John DR, Mittal C, O’Guinn T, Piff P, Roux C, Shah A, Zhu M (2019). The effects of scarcity on consumer decision journeys. Journal of the Academy of Marketing Science.

[CR34] Hemsley-Brown, J. (2022). Antecedents and consequences of brand attachment: A literature review and research agenda. *International Journal of Consumer Studies*, 1–18.

[CR35] Huh YE, Vosgerau J, Morewedge CK (2016). More similar but less satisfying: Comparing preferences for and the efficacy of within- and cross-category substitutes for food. Psychological Science.

[CR36] Inman JJ, Peter AC, Raghubir P (1997). Framing the deal: The role of restrictions in accentuating deal value. Journal of Consumer Research.

[CR37] Jacoby J, Chestnut R (1978). Brand loyalty measurement and management.

[CR38] Ju Y, Jang S (2023). The effect of COVID-19 on hotel booking intentions: Investigating the roles of message appeal type and brand loyalty. International Journal of Hospitality Management.

[CR39] Kahneman D, Knetsch JL, Thaler R (1986). Fairness as a constraint on profit seeking: Entitlements in the market. The American Economic Review.

[CR40] Keller KL (1993). Conceptualizing, measuring, and managing customer-based brand equity. Journal of Marketing.

[CR41] Khamitov M, Wang X, Thomson M (2019). How well do consumer-brand relationships drive customer brand loyalty? Generalizations from a meta-analysis of brand relationship elasticities. Journal of Consumers Research.

[CR42] Khan U, Dhar R, Wertenbroch K, Ratneshwar S, Mick DG (2005). A behavioral decision theoretic perspective on hedonic and utilitarian choice. Inside consumption: Frontiers of research on consumer motives, goals, and desires.

[CR43] Khan U, Zhu M, Kalra A (2011). When trade-offs matter: The effect of choice construal on context effects. Journal of Marketing Research.

[CR44] Kim YK, Sullivan P (2019). Emotional branding speaks to consumers’ heart: The case of fashion brands. Fashion and Textiles.

[CR45] Klein, L. (2020). COVID-19 impact on CPG, *Fractal*.

[CR46] Kleine RE, Kleine SS, Kernan JB (1993). Mundane consumption and the self. Journal of Consumer Psychology.

[CR47] Kristofferson K, McFerran B, Morales AC, Dahl DW (2017). The dark side of scarcity promotions: How exposure to limited-quantity promotions can induce aggression. Journal of Consumer Research.

[CR48] Kwon M, Manikas AS, Barone MJ (2022). (Not) near and dear: COVID-19 concerns increase consumer preference for products that are not “near me”. Journal of the Association for Consumer Research.

[CR49] Labroo AA, Mukhopadhyay A (2009). Lay theories of emotion transience and the search for happiness: A fresh perspective on affect regulation. Journal of Consumer Research.

[CR50] Larsen RJ, Diener E (1987). Affect intensity as an individual difference characteristic: A review. Journal of Research in Personality.

[CR51] Lerner JS, Small DA, Loewenstein G (2004). Heart strings and purse strings: Carryover effects of emotions on economic decisions. Psychological Science.

[CR52] Litt A, Khan U, Shiv B (2010). Lusting while loathing: Parallel counter-driving of wanting and liking. Psychological Science.

[CR53] Liu-Thompkins Y, Khoshghadam L, Shoushtari AA, Zal S (2022). What drives retailer loyalty? A meta-analysis of the role of cognitive, affective, and social factors across five decades. Journal of Retailing.

[CR54] Loureiro S, Ruediger K, Demetris V (2012). Brand emotional connection and loyalty. Journal of Brand Management.

[CR55] Lynn M (1991). Scarcity effects on value: A quantitative review of the commodity theory literature. Psychology & Marketing.

[CR56] MacKinnon DP, Dwyer JH (1993). Estimating mediated effects in prevention studies. Evaluation Review.

[CR57] Mellens M, Dekimpe MG, Steenkamp J-BEM (1996). A review of brand loyalty measures in marketing. Tijdschrift Voor Economie En Management.

[CR58] Miller J (1998). Effects of stimulus–response probability on choice reaction time: Evidence from the lateralized readiness potential. Journal of Experimental Psychology: Human Perception and Performance.

[CR59] Neumann N, Böckenholt U, Ashish S (2016). A meta-analysis of extremeness aversion. Journal of Consumer Psychology.

[CR60] Oskarsson AT, Van Boven L, McClelland GH, Hastie R (2009). What's next? Judging sequences of binary events. Psychological Bulletin.

[CR61] Parker JR, Lehmann DR (2011). When shelf-based scarcity impacts consumer preferences. Journal of Retailing.

[CR63] Sharma E, Alter AL (2012). Financial deprivation prompts consumers to seek scarce goods. Journal of Consumer Research.

[CR64] Shorkey CT, Crocker SB (1981). Frustration theory: A source of unifying concepts for generalist practice. Social Work.

[CR65] Smith CA, Ellsworth PC (1985). Patterns of cognitive appraisal in emotion. Journal of Personality and Social Psychology.

[CR66] Snibbe AC, Markus HR (2005). You can't always get what you want: Educational attainment, agency, and choice. Journal of Personality and Social Psychology.

[CR67] Stauss B, Schmidt M, Schoeler A (2005). Customer frustration in loyalty programs. International Journal of Service Industry Management.

[CR68] Suri R, Kohli C, Monroe KB (2007). The effects of perceived scarcity on consumers’ processing of price information. Journal of the Academy of Marketing Science.

[CR69] Swaminathan A, Sorescu A, Steenkamp JEM, O'Guinn TCG, Schmitt B (2020). Branding in a hyperconnected world: Refocusing theories and rethinking boundaries. Journal of Marketing.

[CR70] Tapscott D, Ticoll D (2003). The naked corporation: How the age of transparency will revolutionize business.

[CR71] Tariton, A. (2020). 24 Things that have been selling out online during the coronavirus pandemic. *USA TODAY*.

[CR72] Thompson DV, Hamilton RW, Banerji I (2020). The effect of childhood socioeconomic status on patience. Organizational Behavior and Human Decision Processes.

[CR73] Thomson M, MacInnis DJ, Park CW (2005). The ties that bind: Measuring the strength of consumers’ emotional attachments to brands. Journal of Consumer Psychology.

[CR74] Tice DM, Bratslavsky E, Baumeister RF (2001). Emotional distress regulation takes precedence over impulse control: If you feel bad, do it!. Journal of Personality and Social Psychology.

[CR75] Tversky A, Kahneman D (1974). Judgment under uncertainty: Heuristics and biases. Science (New Series).

[CR76] Van Herpen E, Pieters R, Zeelenberg M (2009). When demand accelerates demand: Trailing the bandwagon. Journal of Consumer Psychology.

[CR77] Van Steenburg E, Spears N, Fabrize RO (2013). Point of purchase or point of frustration? Consumer frustration tendencies and response in a retail setting. Journal of Consumer Behavior.

[CR78] Verhallen TM, Robben HS (1994). Scarcity and preference: An experiment on unavailability and product evaluation. Journal of Economic Psychology.

[CR79] Wetzer IM, Zelenberg M, Pieters R (2007). Never eat in that restaurant, I did! Exploring why people engage in negative word-of-mouth communication. Psychology and Marketing.

[CR80] Wilson TD, Lisle DJ, Kraft D, Wetzel CG (1989). Preferences as expectation-driven inferences: Effects of affective expectations on affective experiences. Journal of Personality and Social Psychology.

[CR81] Worchel S, Lee J, Adewole A (1975). Effects of sup- ply and demand on ratings of object value. Journal of Personality and Social Psychology.

[CR82] Wu L, Lee C (2016). Limited edition for me and best seller for you: The impact of scarcity versus popularity cues on self vs. other purchase behavior. Journal of Retailing.

[CR83] Zajonc RB, Markus H (1982). Affective and cognitive factors in preferences. Journal of Consumer Research.

[CR84] Zhu M, Ratner RK (2015). Scarcity polarizes preferences: The impact on choice among multiple items in a product class. Journal of Marketing Research.

