# Supplementary material for: Brand loyalty in the face of stockouts
Source: J Acad Mark Sci. 2023 Mar 16:1–31. Online ahead of print. doi: 10.1007/s11747-023-00924-8 (PMC10018630; doi:10.1007/s11747-023-00924-8)
Supplement: Supplementary file 1 — Supplementary file1 (DOCX 1142 kb) [file 11747_2023_924_MOESM1_ESM.docx]

**Web Appendix 1: Bicycle attribute pretest**

This pretest had two goals. The first was to confirm that brands are perceived as relatively affective product attributes. The second was to test a selection of attributes to be used in study 5, and to validate the prediction that some attributes were reliably perceived as having less affective value than brand while others were reliably seen as having more affective value.

**Method**

Participants (N = 141, *M*_age_= 20.6, 62.1% female) were undergraduate students at a U.S. university who completed the study as part of a larger lab session to meet course requirements. They were randomly assigned to condition.

Participants viewed two bicycles (the Affective Upgrade and Non-Affective Upgrade stimuli from study 5, with brand and product order randomized), and were asked to rate the affective value of a series of bicycle attributes: brand, stylish frame and handlebars, customizable color/paint, precision brakes, and shock absorbers on two items: “In general, [this attribute] has…” (1= practical value to 7 = Emotional value) and “I would determine the value of [this attribute] with…” (1 = careful reasoning to 7 = personal feelings). The name of the relevant attribute was substituted into each question.

**Results and discussion**

Results are summarized on Table I. The two measures of affective value were reliably consistent overall (Cronbach’s *α* = .81). We observe that brand was indeed rated as having more affective value than the two non-affective attributes (“precision brakes” and “shock absorbers”) but less affective value than the two affective attributes (“stylish frame and handlebars” and “customizable color paintjob”).

**Table I: Bicycle attribute pretest results for study 5**

|  |  |  | |  | | Comparison to brand | | |
| --- | --- | --- | --- | --- | --- | --- | --- | --- |
| Attribute | **Mean** | | **Standard Error** | | **Cronbach’s**  ***α*** | ***t*** | ***p*** | ***d*** |
| Brand name | 4.19 | | 0.15 | | .82 | ‒ | ‒ | ‒ |
| Stylish frame and handlebars | 5.66 | | 0.12 | | .73 | 7.79 | < .0001 | 0.93 |
| Customizable color paintjob | 6.06 | | 0.10 | | .79 | 10.48 | < .0001 | 1.25 |
| Precision brakes | 1.80 | | 0.11 | | .88 | -12.78 | < .0001 | 1.52 |
| Shock absorbers | 2.05 | | 0.11 | | .92 | -11.54 | < .0001 | 1.37 |

N = 141 observations.

**Web Appendix 2: Durable vs. consumable categories**

**Supplemental results for study 1**

Consumers’ upgrade decisions varied by whether the product was durable (i.e., products which are reused many times and do not require regular replacement) or consumable (i.e., products which are used up quickly and require regular replacement). Running shoes, televisions, and headphones were identified as durable products whereas toilet paper, hand sanitizer, hand soap, rice, and chocolate were identified as consumable products. Durability predicted greater likelihood of upgrading such that 35.28% (SE = 2.72%) of participants tended to prefer option *S+* among durable categories versus 14.74% (SE = 1.45%) among consumable categories (*b* = 1.11, *z*(894) = 5.22, *p* < .0001, odds ratio = 3.05; Model 3-Table II). We believe that these different upgrade patterns in durable and consumable categories are likely due to different expectations of stockouts in these categories during the COVID-19 pandemic rather than due to the natures of the categories themselves. In support of this explanation, we found that participants were less likely to have experienced stockouts of durable products (*b* = -2.26, *z*(896) = -9.98, *p* < .0001, odds ratio = 9.59; Model 1-Table II) and reported greater frustration with such stockouts (*b* = 0.31, *t*(735) = 2.64, *p* < .009, *d* = .03; Model 2-Table II), and these in turn serially mediated the effect of durability on the upgrade decision (5000 bootstrapped mediation; CI [-4.06, -1.08]).

A possible explanation for why consumers might form different expectations of shortages in durable versus consumable categories during the COVID-19 pandemic could arise from the Availability Heuristic (Tversky and Kahneman 1974). For instance, shortages of consumable rather than durable products may have been more available to consumers during the pandemic either because the former were covered more extensively in the media (e.g., toilet paper) or because they were perceived to have clearer logical connections to the pandemic (e.g., hand sanitizer). Likewise, a weekly trip to a grocery store can expose a consumer to empty aisles of toilet paper, soap, or any other everyday consumable items, whereas occasions to witness shortages in durables, such as bicycles, free weights, or tires, may be less frequent. Thus, consumers may have been less likely to expect shortages in durable categories even if those categories did experience pandemic-related supply chain disruptions.

**Table II: Durable versus consumable regression results for study 1**

| Model Number | 1 | 2 | 3 |
| --- | --- | --- | --- |
| Model Type: | Binomial logistic | Linear | Binomial logistic |
| DV: | Experienced stockout | Frustration | Choice of *S+* |
| Predictors |  |  |  |
| Durability | -2.26***  (0.23) | 0.31**  (0.12) | 1.11***  (0.21) |
| Experienced stockout |  | -1.11***  (0.12) | -0.49*  (0.24) |
| Brand preference | -0.96***  (0.16) | 0.73***  (0.11) | 1.11***  (0.20) |
| Frustration |  |  | 0.24***  (0.06) |
| Age | -0.01  (0.01) | 0.00  (0.01) | -0.02  (0.01) |
| Gender (female) | 0.05  (0.17) | 0.36*  (0.16) | -0.62**  (0.22) |
| Employed | 0.13  (0.08) | -0.11  (0.08) | -0.13  (0.10) |
| English | -0.27  (0.56) | -0.90^●^  (0.50) | -0.81  (0.59) |
| Income | 0.00  (0.00) | 0.00  (0.00) | -0.00  (0.00) |
| Urban | 0.33**  (0.13) | 0.00  (0.12) | -0.16  (0.16) |
| Intercept | -1.22^●^  (0.67) | 4.26***  (0.62) | -1.27  (0.81) |
| AIC: | 925.49 | 3549.73 | 833.09 |

N = 909 observations. Standard errors in parentheses. Degrees of freedom for mixed-models calculated using the Satterthwaite Approximation. Significance levels: *p* < .001***, *p* < .010**, *p* < .050*, *p* < .100^●^

**Supplemental results for study 2**

As in study 1, we observed a difference in upgrade behaviors for durable (running shoes, bicycles, televisions, water filter pitchers, coffee makers, and headphones) versus consumable products (remainder of the items). Durability predicted a greater likelihood of upgrading such that 30.53% (SE = 1.45%) of participants tended to prefer *S+* in durable categories versus 18.51% (SE = 1.22%) in consumables categories (*b* = 0.82, *z*(1992) = 5.69, *p* < .0001, odds ratio = 2.27; Table III). Consistent with what we would predict, stockouts of durables engendered greater frustration (*b* = 0.60, *t*(1597) = 6.55, *p* < .0001, *d* = .11; Table III) and this frustration in turn mediated the effect of durability on the upgrade decision (bootstrapped with 5000 iterations; bias corrected 95% CI [1.79, 3.17]). Furthermore, durable products were associated with lower perceptions of demand relating to COVID-19 (*b* = -1.02, *t*(1994) = -47.99, *p* < .0001; *d* = 2.12; *r* = -0.73; Table III).

These results suggest that consumers may have perceived durable categories as unrelated to or unaffected by COVID-19, and that it is this interaction between category and pandemic that drives the results as opposed to an inherent property of durable products. This supposition is supported by study 3 in the main article, which manipulated expectations of shortages in the same category and demonstrated that frustration experienced when expectations are violated caused the effect.

**Table III: Durable versus consumable regression results for study 2**

| Model Number: | 1 | 2 | 3 |
| --- | --- | --- | --- |
| Model Type: | Linear | Binomial logistic | Linear |
| DV: | Frustration | Choice of *S+* | COVID-relevant |
| Predictors |  |  |  |
| Durability | 0.60***  (0.09) | 0.82***  (0.14) | -1.02***  (0.02) |
| COVID-relevant | -0.04  (0.07) |  |  |
| Brand preference | 1.55***  (0.07) | 1.44***  (0.15) | -0.07***  (0.02) |
| Frustration |  | 0.52***  (0.05) |  |
| Age | 0.00  (0.00) | 0.00  (0.01) | -0.00  (0.00) |
| Gender (female) | 0.22*  (0.01) | 0.09  (0.14) | 0.02  (0.02) |
| Employed | 0.01  (0.06) | -0.15^●^  (0.08) | 0.01  (0.01) |
| English | 0.11  (0.28) | 0.40  (0.41) | 0.04  (0.06) |
| Income | -0.00  (0.00) | 0.00  (0.00) | 0.00  (0.00) |
| Urban | -0.07  (0.08) | -0.17^●^  (0.11) | 0.01  (0.02) |
| Intercept | 2.60***  (0.40) | -5.41***  (0.61) | 0.64***  (0.08) |
| AIC: | 7462.85 | 1754.37 | 2754.55 |

N = 2022 observations. Standard errors in parentheses. Degrees of freedom for mixed-models calculated using the Satterthwaite Approximation. Significance levels: *p* < .001***, *p* < .010**, *p* < .050*, *p* < .100^●^
